# A Ligand Selection Strategy Identifies Chemical Probes Targeting the Proteases of SARS‐CoV‐2

**DOI:** 10.1002/anie.202016113

**Published:** 2021-01-28

**Authors:** Lilian Peñalver, Philipp Schmid, Dávid Szamosvári, Stefan Schildknecht, Christoph Globisch, Kevin Sawade, Christine Peter, Thomas Böttcher

**Affiliations:** ^1^ Department of Chemistry Konstanz Research School Chemical Biology Zukunftskolleg University of Konstanz Konstanz Germany; ^2^ In Vitro Toxicology and Biomedicine Department of Biology University of Konstanz Konstanz Germany; ^3^ Albstadt-Sigmaringen University Sigmaringen Germany; ^4^ Department of Chemistry University of Konstanz Konstanz Germany; ^5^ Faculty of Chemistry Department of Biological Chemistry & Centre for Microbiology and Environmental Systems Science Division of Microbial Ecology University of Vienna Vienna Austria

**Keywords:** ABPP, chemical probes, enzyme inhibitors, labeling, proteases

## Abstract

Activity‐based probes are valuable tools for chemical biology. However, finding probes that specifically target the active site of an enzyme remains a challenging task. Herein, we present a ligand selection strategy that allows to rapidly tailor electrophilic probes to a target of choice and showcase its application for the two cysteine proteases of SARS‐CoV‐2 as proof of concept. The resulting probes were specific for the active site labeling of 3CL^pro^ and PL^pro^ with sufficient selectivity in a live cell model as well as in the background of a native human proteome. Exploiting the probes as tools for competitive profiling of a natural product library identified salvianolic acid derivatives as promising 3CL^pro^ inhibitors. We anticipate that our ligand selection strategy will be useful to rapidly develop customized probes and discover inhibitors for a wide range of target proteins also beyond corona virus proteases.

## Graphical abstract

Tuning the ligand space allows to rapidly customize chemical probes for efficacy and active site specificity for a protease of interest. The application of this ligand selection approach is exemplified by generating probes for the two non‐homologous yet essential proteases of the new corona virus SARS‐CoV‐2 providing complementary chemical tools for proteomic labeling and inhibitor discovery.

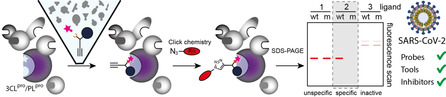

## Introduction

**Figure 1:**
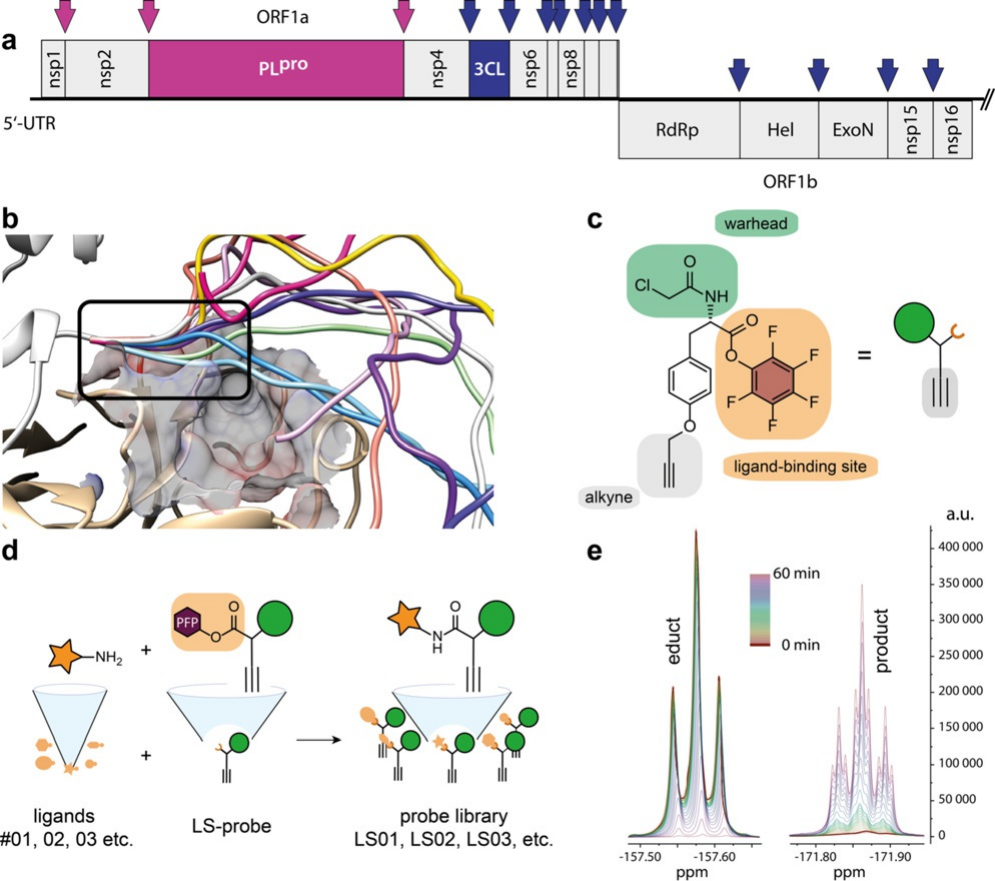
SARS‐CoV‐2 proteases and LS probe strategy. a) Domain architecture of the SARS‐CoV‐2 polyprotein translated from ORF1ab and cleavage sites of the proteases 3CL^pro^ and PL^pro^ liberating the non‐structural proteins (Nsp) from the polyprotein. b) Modeling of the N‐terminus of a dimer of 3CL^pro^. c) Chemical structure and schematic representation of the LS probe with covalent electrophilic warhead, alkyne tag and PFP ester for orthogonal ligand modification. d) Diversification reaction of the LS probe in parallel with individual amine functionalized ligands. e) Kinetics of the diversification reaction with ligand **09** to probe **LS09** followed by ^19^F NMR in 2 min steps for 60 min.

The coronavirus SARS‐CoV‐2 is the etiological agent of the currently unfolding COVID‐19 pandemic. Within less than a year, the disease has infected over 60 million people and caused already more than 1.4 million deaths. In the absence of a global vaccination coverage and adequate therapeutic options the virus is considered to remain a major threat to public health.^[1]^ Worldwide efforts are thus aiming to target proteins essential for the invasion and replication of the virus in the eukaryotic host. In homology to other corona viruses, the first open reading frame (ORF1ab) of the viral RNA genome encodes many proteins required for the replication in the host cell and is translated by a ribosomal frameshifting mechanism. The resulting polyprotein is processed by the viral 3C‐like protease (3CL^pro^) and papain‐like protease (PL^pro^, Figure 1 a).^[2]^ Hence, these proteases are essential for the replication of SARS‐CoV‐2 and therefore prime targets for drug discovery.^[3]^ We thus aimed at targeting these proteases by chemical probes in order to provide tools for tracing the activities of these enzymes and to apply them for the discovery of protease inhibitors. Activity‐based probes are important tools for target and inhibitor discovery and for characterizing the active site reactivity of enzymes in complex proteomes in vitro as well as in live cells.^[4]^ Due to their native reactivity towards electrophiles, proteases have attracted major interest for the development and application of chemical probes.^[5]^ We have recently reported competitive profiling strategies that allow to develop customized enzyme inhibitors, screen synthetic libraries and guide the purification of natural products.^[6]^

Activity‐based probes typically comprise electrophilic warheads in order to covalently bind to a nucleophilic residue in an enzyme's active site. The specificity of a probe for targeting only the active site is achieved by a combination of well‐tuned electrophilicity of its warhead and customized structure to match the steric and electronic demands of the substrate binding pockets. Since most electrophiles readily react also with amino acid residues in the periphery of an enzyme, the major challenge in the development of chemical probes is to customize a probe to exclusively bind to the active site. In order to facilitate the rapid discovery of chemical probes with active site‐specificity, we here report the development of a ligand selection strategy for activity‐based protein profiling (LS‐ABPP) and present the proof of concept by showcasing its application for probe and inhibitor discovery for the two proteases of SARS‐CoV‐2.

## Results and Discussion

**Protease activity**. Both 3CL^pro^ and PL^pro^ liberate themselves from the polyprotein by cleaving their respective N‐ and C‐terminal sequences as described for the homologous sequences of SARS‐CoV‐1.[^3a^, ^7^]

Hereby dimerization and maturation of 3CL^pro^ by N‐terminal self‐cleavage is required for full activation of the protease and the ability of trans‐processing of other non‐structural proteins.^[8]^ Dimeric crystal structures of mature 3CL^pro^ show that the N‐terminus of one 3CL^pro^ monomer is in close proximity to the active site pocket of the other monomer and the N‐terminus is cleaved between the short consensus sequence Leu–Gln and Ser already during expression.^[9]^ Molecular modeling of the flexible peptide sequence prior to cleavage indicated that the first amino acids before the cleavage site occupy a part of the active site pocket (Figure 1 b), which is in agreement with mechanistic models of protease maturation.^[8]^ The importance of a cleaved N‐terminus for full enzymatic activity has also been previously described for 3CL^pro^ of SARS‐CoV.^[10]^ We reasoned that inhibitors targeting the protease prior to full activation could be of great value for drug development against SARS‐CoV‐2. Thus, we constructed a 3CL^pro^ version fused with a non‐cleavable N‐terminal Strep‐tag II (t3CL^pro^). Modeling predicted an identical behavior of the fusion peptide at the active site compared to the native sequence of wild type prior to cleavage (Supporting Information Figure S1). Codon‐optimized sequences of the 3CL^pro^ and PL^pro^ domains of nsp3 and nsp5 of SARS‐CoV‐2 were ultimately cloned into an IPTG inducible vector and heterologously expressed in *Escherichia coli*. Proteins were either purified via affinity chromatography or used in situ in the native cell of the expression system. Confirming our predictions regarding the N‐terminal modification, the purified t3CL^pro^ version indeed was inactive in a protease assay using oligopeptide substrates linked to a fluorogenic 7‐amino‐4‐methylcoumarin group (Supporting Information Figure S2). This represented the unique opportunity to validate the utility of the LS‐ABPP strategy against PL^pro^ and the pre‐activation stage of 3CL^pro^ of SARS‐CoV‐2 and demonstrate its versatility for customizing probes to different targets.

**LS‐ABPP allows to rapidly screen the ligand space**. We designed LS‐ABPP to rapidly tailor a probe scaffold to the active site pocket of an enzyme of interest and demonstrate its potential for the development of specific probes against t3CL^pro^ and PL^pro^ of the pandemic corona virus SARS‐CoV‐2. We synthesized a chemical LS probe based on the core of tyrosine. The probe was equipped with a chloroacetamide warhead conferring reactivity towards the protein's active site and a terminal alkyne group for fluorescent labeling of probe‐bound enzyme by bioorthogonal click chemistry via the 1,3‐dipolar Huisgen alkyne–azide cycloaddition (Figure 1 c). As the central element of the LS probe concept, a pentafluorophenyl (PFP) ester was installed to allow the rapid orthogonal diversification of the probe scaffold by the reaction with ligand libraries of primary or secondary amines (Figure 1 d). The LS probe was reacted individually (in parallel) with a selection of 27 amine‐containing ligands in microliter scale (Supporting Information Table S1). Time‐resolved ^19^F NMR spectra helped to optimize the reaction conditions and revealed complete conversion after 60 min even for the least reactive aromatic amines (Figure 1 e). Primary aliphatic amines reacted within minutes, which was too fast to track by NMR spectroscopy (Supporting Information Figure S3). Although a quantitative reaction was expected, potential residues of unreacted probe were removed using amino‐functionalized polystyrene beads to prevent unspecific protein reactivity. Subsequently, the reaction mixture was lyophilized and dissolved in DMSO before direct application to protein labeling experiments.

**Figure 2:**
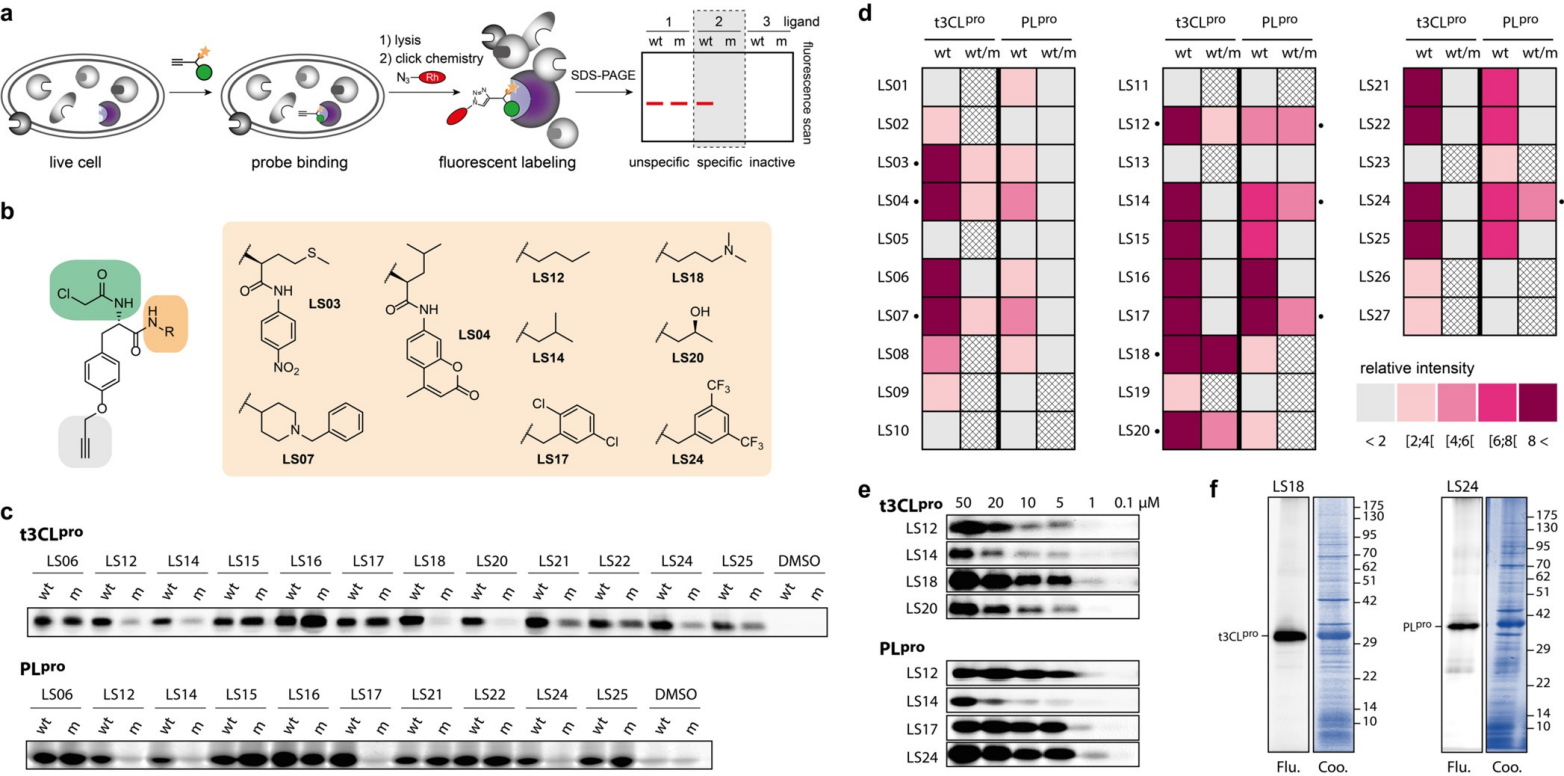
Specific and selective labeling of SARS‐CoV‐2 proteases. a) Live cell labeling of target proteins in *E*. *coli*. Cells expressing t3CL^pro^ and PL^pro^ were treated with the probes followed by cell lysis and click chemistry to append a fluorescent reporter tag and subsequent gel electrophoresis. b) Structures of members of the ligand‐diversified LS probe library that showed the most potent and specific effects. c) Example for the ligand selection screening with live cell labeling of wild type (wt) and active site mutant (m) by the LS probe library at 20 μm. Representative data of three independent replicates. d) Quantification of wild type labeling intensities (wt) normalized to DMSO controls and specificity (wt/m) of LS probes at 20 μm. Specific probes exhibit a high wild type to mutant ratio (*n*=3). Criss–cross pattern: not determined. e) Concentration series of the most specific probes for the labeling of t3CL^pro^ and PL^pro^. f) Selectivity of probes **LS18** and **LS24** at 20 μm in live cell labeling of their overexpressed target proteases in the background of a native *E*. *coli* proteome. Flu: fluorescence gels; Coo: Coomassie stained gels. Representative gels of three independent replicates.

**Ligand selection confers specificity for active site labeling**. In order to discover probes with specificity for the active site of the proteases, we constructed the corresponding active site mutants Cys145Ala (t3CL^pro^) and Cys114Ala (PL^pro^). We then screened the probes in situ against t3CL^pro^ and PL^pro^ expressed in intact *E*. *coli* cells and their corresponding active site mutants (Figure 2 a). To this aim we incubated the cells with 20 μm of the ligand‐modified probes (**LS01**–**LS29**) for one hour (Figure 2 b), followed by cell lysis and click chemistry with tetramethylrhodamine (TAMRA) azide to append a fluorescent reporter tag. After gel electrophoresis by SDS‐PAGE, fluorescence imaging revealed probe labeling of the enzymes (Figure 2 a). Some of the probes resulted in strongly labeled bands of t3CL^pro^ and PL^pro^ while other probes were inactive regarding one or both proteases. Probes that displayed considerable labeling intensity were further examined for their specificity by comparing the labeling of wild type (wt) versus active site mutant (m) proteins (Figure 2 c,d; Supporting Information Figure S4). Fluorescence intensities were quantified relative to the DMSO control and probes with a ratio wt/m>2 were considered specific. Most probes did not discriminate for active site labeling or were inactive, which underscores the challenge of finding active site‐specific binders. However, we could identify several probes that exhibited great specificity for labeling only the wild type but not mutant t3CL^pro^ and PL^pro^ (Figure 2 d). The enzymes exhibited preferences for different ligand‐modified LS probes. For example, probes **LS18** and **LS20** showed specificity for the labeling of t3CL^pro^ but not PL^pro^ while **LS12**, **LS14**, **LS17**, and **LS24** were specific for PL^pro^ but not for t3CL^pro^.

It was thus possible to identify complementary probes for the two proteases. To estimate their sensitivity, we performed labeling experiments with the most specific probes in concentration dependence. Strikingly, t3CL^pro^ was labeled by **LS18** and PL^pro^ by **LS17** and **LS24** as the most sensitive probes at concentrations as low as 1 μm (Figure 2 e). Interestingly, overproduced t3CL^pro^ and PL^pro^ were the only bands that probes **LS18** and **LS24** labeled in live *E*. *coli* cells, emphasizing the selectivity of the probes in the background of a native proteome (Figure 2 f).

**Figure 3:**
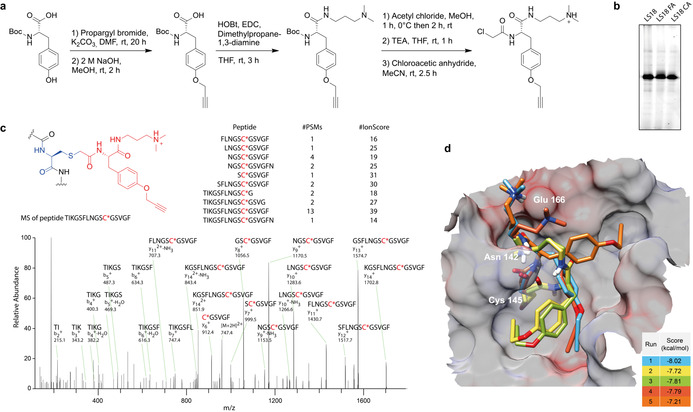
Active site‐directed mechanism of probe **LS18**. a) Direct preparative synthesis of **LS18** as chloroacetate (**LS18 CA**) and formate (**LS18 FA**) salts. b) Labeling of t3CL^pro^ in live cells of *E*. *coli* using in situ produced **LS18** in comparison with purified synthetic **LS18 CA** and **LS18 FA** at 20 μm each. c) Protein sequencing of **LS18**‐labeled t3CL^pro^ by mass spectrometry revealed covalent modification of the active site cysteine. Representative results of two independent replicates. d) Molecular docking of **LS18** into the active site of the crystal structure of 3CL^pro^ (PDB ID: 6YB7). A movie of the best‐scoring docking poses of **LS18** is available in the Supporting information.

**LS18 binds covalently to the active site cysteine of t3CL^pro^**. In order to exemplarily validate the identity and activity of ligand‐modified LS probe, we developed a preparative synthesis route towards probe **LS18** (Figure 3 a). In short, starting from Boc‐protected l‐tyrosine, the alkyne was installed by etherification with propargyl bromide and the ligand moiety was introduced by amide coupling with *N*,*N*‐dimethylpropane‐1,3‐diamine. Lastly, the chloroacetamide warhead was mounted yielding **LS18** as chloroacetate and formiate salts. Compared to in situ reacted **LS18** by LS probe diversification, both synthetic versions of the probe showed identical activity and selectivity in the native *E*. *coli* proteome (Figure 3 b). In order to confirm the active site‐directed mode of action and identify the site of covalent modification, t3CL^pro^ was labeled in live *E*. *coli* cells with probe **LS18** followed by cell lysis and SDS‐PAGE without click chemistry. The Coomassie‐stained band of t3CL^pro^ was cut out, digested with pepsin, and subjected to sequencing by mass spectrometry. Interrogating amino acid modifications by **LS18** identified the active site Cys145 as the site of covalent attachment of the probe (Figure 3 c).

Building on these results we performed covalent molecular docking of **LS18** into the active site of the crystal structure of t3CL^pro^ of SARS‐CoV‐2 (PDB ID: 6YB7).^[9a]^ Although the five best scoring docking results show some diversity, the general orientation of the probe was similar in all cases (Figure 3 d). The molecular docking of **LS18** gave similar results for 3CL^pro^ in its native form with truncated N‐terminus as well as with our structural models of the extended native N‐terminus prior to self‐cleavage of 3CL^pro^ and the Strep‐tag II‐modified non‐cleavable N‐terminus (Supporting Information Figure S5). To exclude an influence of the N‐terminal Strep‐tag II for probe binding, we used an enterokinase cleavage site to remove the tag, yielding a version of 3CL^pro^ only two amino acids longer than the native N‐terminus after self‐cleavage. Successful labeling of this truncated version of t3CL^pro^ with probe **LS18** confirmed that the small size of the probe allowed to profile the active site of the protease independent from its activation stage (Supporting Information Figure S6).

**Comparing homologs of 3CL^pro^**. In order to investigate if our LS probe strategy also allows to label 3CL^pro^ of the closely related SARS‐CoV‐1, another member of the *Sarbecovirus* subgenus, we also expressed its wild type and mutant sequences and performed LS probe labeling experiments in situ.

For most ligand‐modified LS probes the labeling intensity and specificity for the active site of both t3CL^pro^ homologs were virtually identical (Supporting Information Figure S7a), demonstrating robustness even across different viral strains. However, probes **LS06** and **LS17** displayed concentration‐dependent specificity for t3CL^pro^ of SARS‐CoV‐1 while being unspecific for SARS‐CoV‐2 (Supporting Information Figure S7b–d). These minor yet important differences highlight the possibility of using the LS probe strategy for the fine tuning of chemical probes to the active site properties of an enzyme of interest.

**Figure 4:**
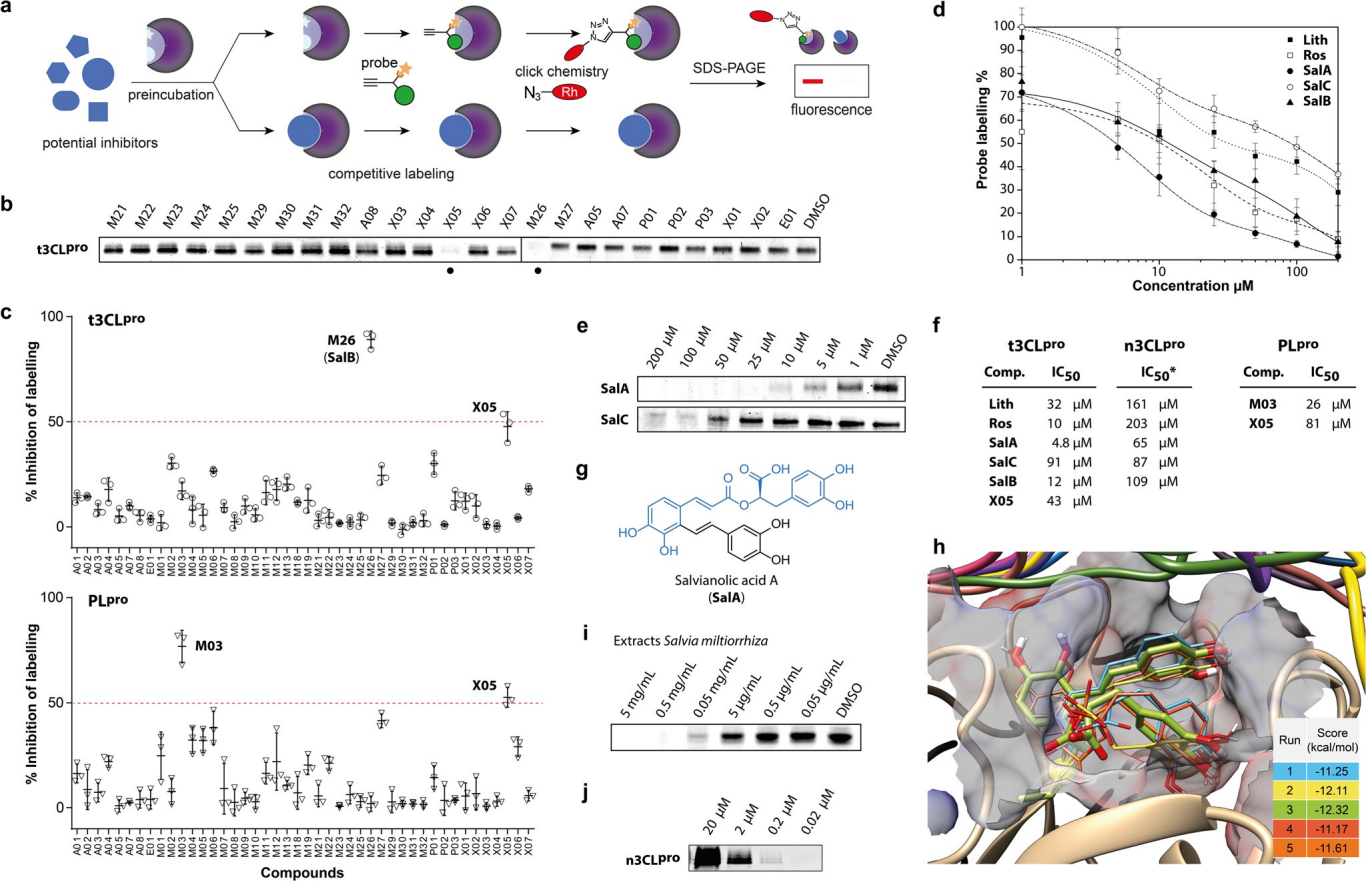
Application of chemical probes for protease labeling and competitive profiling. a) Competitive profiling strategy where the proteases are preincubated with potential inhibitors followed by competitive labeling with a specific probe. Fluorescence in SDS gels is abolished or reduced in the case of successful inhibition of the active site. b) Example of competitive labeling of 3CL^pro^ with members of the compound library at 200 μm and probe **LS18** at 5 μm. c) Results of the initial competitive screening of the library electrophilic compounds at 200 μm against 3CL^pro^ (**LS18**) and PL^pro^ (**LS24**) (*n*=3). d) Inhibition curves of 3CL^pro^ inhibitors determined by quantification of the fluorescent labeling intensity by **LS18** (*n*=3). **Lith**: lithospermic acid; **Ros**: rosmarinic acid; **SalA**: salvianolic acid A; **SalC**: salvianolic acid C; **SalB**: salvianolic acid B. e) Examples of fluorescent gels with competitive labeling of 3CL^pro^. f) IC_50_ values of the most active inhibitors quantified by competitive fluorescence labeling. g) Chemical structure of salvianolic acid A (**SalA**) with the common structure motif colored in blue. h) Covalent docking of **SalA** into the active site of t3CL^pro^ (PDB ID: 6YB7). i) Competitive labeling with concentration series of extracts of the roots of *Salvia miltiorrhiza*. j) Labeling of mature n3CL^pro^ (0.2 mg mL^−1^) by **LS18** in dependence of the probe concentration.

**Competitive screening identifies enzyme inhibitors**. Since our probes exhibited sensitive and active site‐specific labeling of PL^pro^ as well as of the pre‐activation stage of 3CL^pro^, we aimed to exploit their potential as chemical tools for the competitive screening of enzyme inhibitors. We searched over 1000 structures of commercially available food grade additives, natural products, and protease inhibitors, most of which were approved for human use, and manually selected 44 compounds with electrophilic motifs such as aldehydes, Michael acceptors, epoxides, and esters that could potentially react covalently with the nucleophilic active site cysteine of 3CL^pro^ and PL^pro^ (Supporting Information Table S2). The electrophilic compound library was prepared in form of DMSO stocks and individually pre‐incubated for an initial screening with the purified proteases followed by labeling with the probes (**LS18** for t3CL^pro^ and **LS24** for PL^pro^). Successful inhibitors would block the active site and thereby prevent subsequent probe labeling, which could be read out by lowered in‐gel fluorescence (Figure 4 a). Indeed, at an initial concentration of 200 μm some compounds even abolished probe labeling (Figure 4 b, Supporting Information Figure S8). We then quantified the fluorescence signal relative to the control and compounds that resulted in more than 50 % inhibition of competitive labeling were selected to investigate their dose–response relationship (Figure 4 c). Half‐maximal inhibitory concentrations (IC_50_s) were calculated from curve fittings of concentration‐dependent quantitative competitive labeling (Figure 4 d).

Phenethyl isothiocyanate, which is produced from its precursor gluconasturtiin by vegetables of the *Brassicaceae* family,^[11]^ scored against both proteases likely due to unspecific thiol reactivity of isothiocyanates, but only led to incomplete inhibition (**X05**, Supporting Information Figure S9a,b). In contrast, curcumin (**M03**) almost completely inhibited labeling of PL^pro^ with an IC_50_ of 26 μm and was inactive against t3CL^pro^ (Supporting Information Figure S9b). However, curcumin is a well‐known pan‐assay interference (PAIN) compound and as such of no particular interest.^[12]^ Both, **X05** and **M03** inhibited enzyme activity of PL^pro^ with a fluorogenic peptide substrate, confirming the validity of the competitive screening approach (Supporting Information Figure S10). Of greater interest was the activity of salvianolic acid B (**M26**, **SalB**), which inhibited labeling of t3CL^pro^ with an IC_50_ of 12 μm (Figure 4 d–f). We thus focused on compounds with a closely related caffeic acid ester motif. All compounds showed a clear dose–response behavior whereby rosmarinic acid (**Ros**) and salvianolic acid A (**SalA**) were the most active with IC_50_ values of 10 and 4.8 μm, respectively. Interestingly, salvianolic acid C (**SalC**), which differs from **SalA** only by a hydroxy group locked into a benzofuran ring, was considerably less active with an IC_50_ of 91 μm (Figure 4 e–g). Also, the closely related lithospermic acid (**Lith**) only exhibited an IC_50_ of 32 μm. These results demonstrate a fine‐tuned structure–activity relationship of salvianolic acid derivatives for the inhibition of t3CL^pro^. To achieve a better understanding of the molecular basis of protease inhibition, we performed molecular docking of **SalA** into the active site of t3CL^pro^ (Figure 4 h). All of the best docking results showed almost identical orientations in the active site with multiple hydrogen bond contacts via the cresol moieties and considerably higher docking scores than for the **LS18** probe. **SalA** filled most of the active site pocket and docking was not obstructed by the N‐terminal modification, suggesting that competitive labeling with customized LS probes allows to discover inhibitors that can block 3CL^pro^ even in its pre‐activation stage. Since **SalA** and the other potent salvianolic acid derivatives are produced by the plant *Salvia miltiorrhiza* (red sage), we finally prepared and tested extracts of its dried roots in a competitive labeling assay with probe **LS18** against t3CL^pro^ and observed potent inhibition down to 1 mg mL^−1^ (Figure 4 i). To investigate the effect of the N‐terminus of the pre‐activation stage of 3CL^pro^ on probe and inhibitor binding, we performed additional experiments with native 3CL^pro^ (n3CL^pro^) comprising a cleaved N‐ and C‐terminus. Probe **LS18** labeled n3CL^pro^ with great sensitivity at probe concentrations down to 0.2 μm (Figure 4 j). In contrast to t3CL^pro^, the fully maturated n3CL^pro^ showed activity in processing fluorogenic substrates (Supporting Information Figure S11). Consequently, we employed substrate cleavage assays to determine the IC_50_ values for the inhibitors identified for the pre‐activation stage of 3CL^pro^. All salvianolic acid‐related compounds completely inhibited the enzymatic activity of n3CL^pro^ albeit at comparably high concentrations (Figure 4 f, Supporting Information Figure S12). For example, **SalA** gave an IC_50_ of 65 μm for the inhibition of n3CL^pro^. Conversely, GC376,^[13]^ a known nanomolar inhibitor of mature 3CL^pro^, comparably weakly reduced t3CL^pro^ labeling in a competition experiment (Supporting Information Figure S13).

Our results suggest that inhibitors of the pre‐activation stage of 3CL^pro^ encompass somewhat different structural requirements than inhibitors of mature 3CL^pro^ and may be missed in standard screening assays.

**Figure 5:**
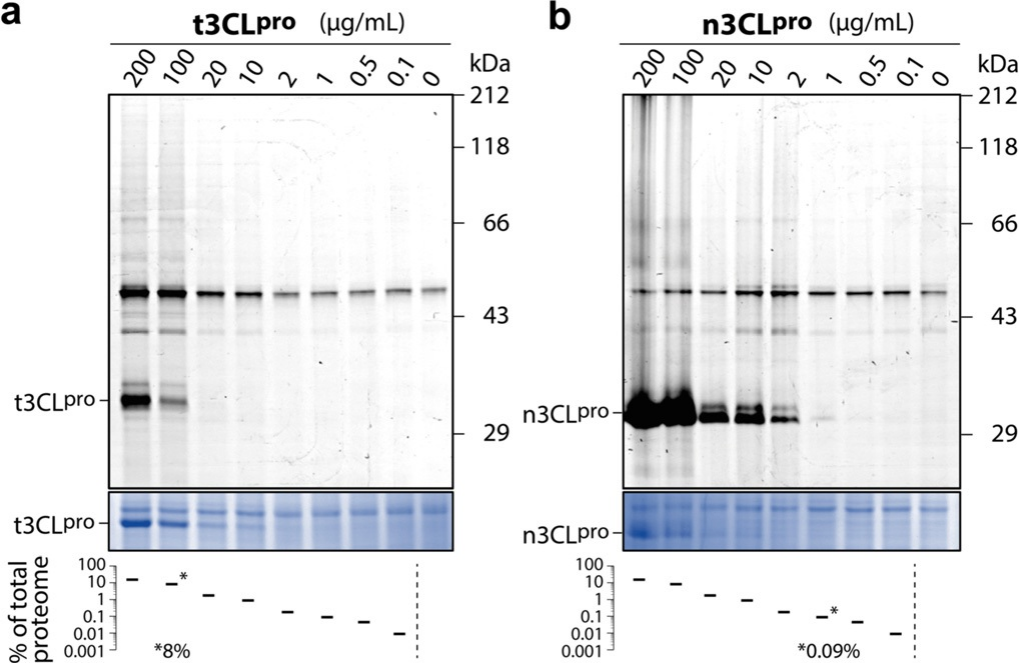
Detection of 3CL^pro^ titrated into the background of a native proteome (1.14 mg mL^−1^) of A549 human alveolar epithelial cell lysates. a) Labeling of t3CL^pro^ and b) n3CL^pro^ by probe **LS18** at 20 μm probe concentration. The percentages of supplemented 3CL^pro^ are given in relation to the total proteome. The absolute value is indicated for the corresponding detection limit.

**Protease labeling in the human proteome**. We next aimed to investigate a potential application of the probes to label the proteases in the background of a native human proteome. We used lysates of human hepatocellular carcinoma (HepG2) cells and supplemented them with different concentrations of purified t3CL^pro^ and PL^pro^. Applying probe **LS18** for t3CL^pro^ and **LS24** for PL^pro^ at 20 μm followed by click chemistry resulted in the detection of each of the proteases as strong bands at protease concentrations down to 77 μg mL^−1^ (Supporting Information Figure S14). Finally, we compared the labeling of t3CL^pro^ and n3CL^pro^, titrated into the proteome of human alveolar epithelial cells (A549) that had been used previously in infection models of SARS‐CoV‐2.^[14]^ While t3CL^pro^ was labeled by probe **LS18** with equal sensitivity as in the HepG2 proteome (Figure 5 a), n3CL^pro^ could even be detected at concentrations of 1 μg mL^−1^, the proportion of n3CL^pro^ in the native proteome being only 0.09 % (Figure 5 b).

Only relatively few additional off‐target bands were labeled in eukaryotic cell lysates, which suggests that these probes could additionally be employed to label and detect the activity of SARS‐CoV‐2 proteases in the background of a complex proteome.

The proteases of the SARS‐CoV‐2 are currently in the focus of research for drug development against COVID‐19.[^9b^, ^15^] Although activity‐based probes represent important chemical tools for basic research and drug discovery, there has been surprisingly little work on corona viruses so far. Only a peptidic substrate‐based probe for 3CL^pro^ has been described recently.^[16]^ We have developed a ligand selection strategy that allows to rapidly tailor chemical probes for the active site‐specific labeling of an enzyme of interest and demonstrated its versatility for finding activity‐based probes against the corona virus proteases 3CL^pro^ and PL^pro^. While all probes featured an identical electrophilic chloroacetamide warhead, only few of them exhibited high‐level specificity by only labeling the nucleophilic active site cysteine. These results show that target specificity depends on choosing the right ligand and suggest that extensive ligand screening is key to finding optimized chemical probes. Our ligand selection approach allows to rapidly screen structural diversity in the ligand site resulting in optimized active site‐specific probes without the need for the time‐demanding syntheses of larger probe libraries. Activity‐based probes have been useful to functionally characterize enzymes and profile their activity in complex proteomes.^[17]^ We anticipate that our 3CL^pro^‐ and PL^pro^‐specific probes will provide important tools to further investigate the activity function of these important corona virus proteases. These may be of particular interest for studying the activation of 3CL^pro^ by self‐cleavage or the deubiquitinating (DUB) activity of PL^pro^ that modulates proteostasis of the host cell during the infection process.^[18]^ Furthermore, we have demonstrated that our chemical probes are useful tools for inhibitor discovery against 3CL^pro^ and PL^pro^ by competitive profiling. A great amount of work has been dedicated to finding inhibitors of the mature stage of 3CL^pro^ using virtual^[19]^ and substrate‐based screening approaches[^9b^, ^15b^, ^20^] as well as rational design and structure‐guided optimization approaches.[^15a^, ^21^] These recent developments could also benefit from the vast amount of previous work on 3CL^pro^ inhibitors of SARS‐CoV.[^3a^, ^22^] Our 3CL^pro^‐specific probes provide the unique opportunity to study this protease prior to N‐terminal self‐cleavage and discover inhibitors of a pre‐maturation stage of the enzyme that cannot be assessed using conventional substrate cleavage assays. We identified salvianolic acid derivatives as potent inhibitors of 3CL^pro^, with salvianolic acid A effectively blocking probe labeling at single‐digit micromolar concentrations. Also, extracts of roots of *Salvia miltiorrhiza* (red sage), the natural producer of salvianolic acids, confirmed a strong inhibitory activity. Extracts of *S. miltiorrhiza* and **SalA** are known for having anti‐thrombotic effects^[23]^ and salvianolate injections (mostly **SalB**) have been clinically approved and are widely used in China for the treatment of coronary heart disease.^[24]^ Since cardiovascular diseases are one of the greatest risk factors for COVID‐19‐caused mortality and morbidity,^[25]^ cardiovascular drugs with additional activity as corona virus protease inhibitors would be of major interest. Our results are thus of immediate relevance for SARS‐CoV‐2 research and drug development, and our novel ligand selection strategy is of general importance for designing tools for enzymology and chemical proteomics.

## Conclusion

We report the development of a highly adaptable ligand selection strategy that allows to rapidly customize chemical probe scaffolds in order to achieve active site specificity for a protease of interest. As proof of concept we targeted the two cysteine proteases of the corona virus SARS‐CoV‐2, demonstrating that selectivity and efficacy can be indeed controlled and fine‐tuned via the ligand structure. We found probes with single‐digit micromolar activity for corona virus 3CL^pro^ and PL^pro^ proteases and highlighted their value as chemical tools for the active site‐specific labeling of these proteases in complex proteomes as well as for the screening of inhibitors for drug development against COVID‐19.

## Conflict of interest

The authors declare no conflict of interest.

## Supporting information

As a service to our authors and readers, this journal provides supporting information supplied by the authors. Such materials are peer reviewed and may be re‐organized for online delivery, but are not copy‐edited or typeset. Technical support issues arising from supporting information (other than missing files) should be addressed to the authors.

Supplementary
