# Supplementary material for: A Ligand Selection Strategy Identifies Chemical Probes Targeting the Proteases of SARS‐CoV‐2
Source: Angew Chem Int Ed Engl. 2021 Jan 28;60(12):6799–806. doi: 10.1002/anie.202016113 (PMC7986205; doi:10.1002/anie.202016113)
Supplement: Supplementary file 1 — Supplementary [file ANIE-60-6799-s001.pdf]

Angewandte Chemie
Eine Zeitschrift der Gesellschaft Deutscher Chemiker

Supporting Information

A Ligand Selection Strategy Identifies Chemical Probes Targeting the Proteases of SARS-CoV-2

*Lilian Peñalver, Philipp Schmid, Dávid Szamosvári, Stefan Schildknecht, Christoph Globisch, Kevin Sawade, Christine Peter, and Thomas Böttcher**

anie_202016113_sm_miscellaneous_information.pdf

1. Syntheses 2
1.1. Synthesis of LS-Probe 2
1.2. Preparative synthesis of probe LS18 3
2. Recombinant proteins 6
2.1. Plasmid preparation 6
2.2. Transformation of plasmids 9
2.3. Overproduction of proteins 10
2.4. Affinity-purification of proteins 10
2.5. Enterokinase digest of t3CL^{pro} 10
3. Probe reactions 10
3.1. LS-probe modification with ligands 10
3.2. *In situ* probe labelling of proteins in live *E. coli* cells 10
3.3. *In vitro* probe labelling of proteins in lysates and purified protein 11
3.4. LS-probe labelling of virus proteases in background of A549 and HepG2 proteomes 11
3.5. Competitive profiling experiments 11
3.5.1. *In situ* competitive profiling 11
3.5.2. In vitro competitive profiling 11
3.6. Click reactions 12
3.7. SDS-PAGE and in-gel fluorescence scanning 12
4. Proteomic experiments 12
5. Substrate-based enzyme inhibition assays 12
6. Preparation of root extracts from red sage (*Salvia miltiorrhiza*) 13
7. Molecular docking 13
References 14
Tables 15
Figures 19
Annex: NMR Spectra 33

1. Syntheses

1.1. Synthesis of LS-Probe

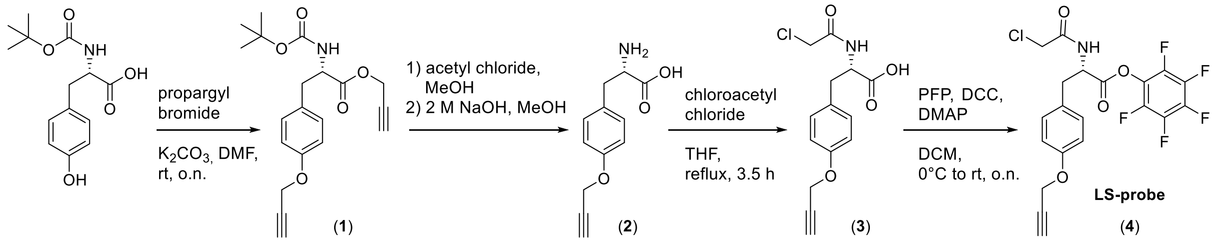

2-*tert*-Butoxycarbonylamino-3-[4-(prop-2-ynyloxy)phenyl]-propionic acid propargyl ester (1)

The reaction was adapted from a protocol described in the literature[1]. 6 g of (21.3 mmol, 1 eq) *N-tert*-Butoxycarbonyl-tyrosine and 9 g of (56.1 mmol, 3 eq) K_2CO_3 were suspended in 30 mL dry DMF under N_2 flow. After stirring for 10 min at room temperature 7.9 mL (73.1 mmol, 3.5 eq) of an 80 % solution propargyl bromide in toluene was slowly added. The solution was left to react for 18 h at room temperature. 150 mL H_2O were used to quench the reaction. The mixture was extracted with diethyl ether, washed with distilled water and brine. The combined organic layers were dried over $MgSO_4$ before solvent evaporation *in vacuo*. The yellow oil was used in the next step without any further purification (7.3 g, 100 %). R_f = 0.63 (petrol ether/ethyl acetate 1:1).
^{1}H-NMR ($CDCl_3$, 400 MHz) δ (ppm): 1.42 (s, 9 H, (C$\underline{H_3}$)$_3$C-), 2.51 (m, 2 H, -$CO_2CH_2CC\underline{H}$, -$PhOCH_2CC\underline{H}$), 3.07 (m, 2 H, -PhC$\underline{H_2}$CH-), 4.59-4.93 (m, 1 H, -PhCH$_2$C$\underline{H}$-), 4.64-4.80 (m, 2 H, -CO$_2$C$\underline{H_2}$CCH, -PhOC$\underline{H_2}$CCH), 6.90 (m, 2 H, aromat.), 7.09 (m, 2 H, aromat.)

2-Amino-3-[4-(prop-2-ynyloxy)phenyl]-propionic acid propargyl ester (1b)

The reaction was adapted from a protocol described in the literature[1]. To 180 mL of MeOH on an ice bath, 21 mL (294 mmol, 15 eq) acetyl chloride were slowly added. The solution was left to stir for 10 min at 0 °C. 7 g of **(1)** were added and the solution was allowed to warm to room temperature. After 2 h the solvent was evaporated *in vacuo* to give the pure product as slightly brownish-white powder (4.1 g, 100 %).
^{1}H-NMR (D_2O, 400 MHz) δ (ppm): 2.98 (t, J = 2.4 Hz, 1 H, -OCH$_2$CC$\underline{H}$), 3.30-3.09 (m, 2 H, -PhC$\underline{H_2}$CH-), 3.99 (dd, 1 H, J = 7.8, J = 5.2 Hz, -PhCH$_2$C$\underline{H}$-), 4.85 (d, 2 H, J = 2.4 Hz, -OC$\underline{H_2}$CCH), 7.10 (m, 2 H, aromat.), 7.33 (m, 2 H, aromat.).

***O*-Propargyl tyrosine (2)**

The reaction was adapted from a protocol described in the literature[1]. To a mixture of 30 mL MeOH and 42 mL 2M NaOH 5.6 g (20 mmol, 1 eq) **(1b)** was added. The reaction was stirred for 17 h at room temperature. With concentrated HCl the pH of the mixture was adjusted to 7 and it was kept at 4 °C for 4 h. The precipitate was filtered off and dried *in vacuo* to give the pure product as light yellow powder (3.51 g, 17.1 mmol, 83 %).
^{1}H-NMR (D_2O, 400 MHz) δ (ppm) 2.98 (m, 1 H, -PhOCH$_2$CC$\underline{H}$), 3.12 (dd, 1 H, J = 14.9, J = 8.1 Hz, -PhC$\underline{H_2}$CH-), 3.27 (dd, 1 H, J = 14.3, J = 4.9 Hz, -PhC$\underline{H_2}$CH-), 4.00 (td, 1 H, J = 13.1, J = 2.2 Hz, -PhCH$_2$C$\underline{H}$-), 4.85 (t, 2 H, J = 1.7 Hz, -PhOC$\underline{H_2}$CCH), 7.11 (m, 2 H, aromat.), 7.32 (m, 2 H, aromat.).

2-(2-Chloroacetamido)-3-(4-(prop-2-yn-1-yloxy)phenyl)propanoic acid (3)

The reaction was adapted from a protocol described in the literature[2]. 1.03 g (5 mmol) of **(2)** were suspended in 35 mL of dry THF under an N_2 flow. 0.6 mL (1.5 eq) of chloroactyl chloride were added. The suspension was stirred at reflux for 3 hours. The reaction was extracted with ethyl acetate and washed with distilled water and brine. The combined organic layers were dried over $MgSO_4$ before solvent evaporation *in vacuo*. The yellow crystals were purified via column chromatography (DCM:MeOH=9:1) to give the pure product as light yellow crystals (1.19 g, 85 %). R_f = 0.54 (petrol ether/ethyl acetate 1:1).
^{1}H-NMR ($CDCl_3$, 400 MHz) δ (ppm): 2.52 (t, 1 H, *J* = 2.2 Hz, -OCH$_2$CC<u>H</u>), 3.09-3.23 (m, 2 H, -PhC<u>H</u>$_2$CH-), 4.05 (d, *J* = 2.0 Hz, 2 H, -OC<u>H</u>$_2$CCH), 4.68 (d, *J* = 2.5 Hz, 2 H, -C<u>H</u>-Cl$_2$), 4.86 (m, 1 H, -PhCH$_2$C<u>H</u>-), 6.94 (d, 2 H, *J* = 8.6 Hz, aromat.), 7.11 (d, *J* = 8.4 Hz, 2 H, aromat.).
ESI-HRMS: m/z = 296.0685 $[M+H]^+$, calc. for $C_{14}H_{14}ClNO_4 + H^+$ = 296.0684.

Perfluorophenyl 2-(2-chloroacetamido)-3-(4-(prop-2-yn-1-yloxy)phenyl)propanoate (4, LS-probe)

The reaction was adapted from a protocol described in the literature[3]. 0.92 g (3.2 mmol) of **(3)** and 0.72 g (3.6 mmol) of pentafluorophenol were dissolved in 80 mL dry DCM under nitrogen. The solution was stirred on ice for 20 min before adding 40 mg of DMAP (0.32 mmol) and 0.74 g DCC (3.6 mmol). The suspension was stirred over night at room temperature. The reaction was quenched with 4 mL 3N HCl. The resulting solution was kept at 4 °C over night. The precipitate was filtered off over celite and washed with cold DCM. Afterwards the filtrate was washed with saturated $NaHCO_3$ solution and distilled water, dried over $MgSO_4$ before the solvent was evaporated *in vacuo*. The solid was purified by flash chromatography (ethyl acetate, hexane) to give 0.93 g (63 %) of the pure product as pale yellow crystals. R_f = 0.88 (ethyl acetate).
^{1}H-NMR ($CDCl_3$, 400 MHz) δ (ppm):, 2.51 (t, 1 H, *J* = 1.8 Hz, -OCH$_2$CC<u>H</u>), 3.11-3.23 (m, 2 H, -PhC<u>H</u>$_2$CH-), 4.05 (d, *J* = 4.5 Hz, 2 H, -OC<u>H</u>$_2$CCH), 4.68 (d, *J* = 2.1 Hz, 2 H, -C<u>H</u>-Cl$_2$), 4.87 (dt, *J* = 6.32, *J* = 7.1 Hz, 1 H, -PhCH$_2$C<u>H</u>-), 6.94 (d, 2 H, *J* = 8.5 Hz, aromat.), 6.96 (s, 1 H, -NH-), 7.11 (d, *J* = 8.7 Hz, 2 H, aromat.).
^{13}C-NMR ($CDCl_3$, 400 MHz) δ (ppm): 36.93 (Ph<u>C</u>H$_2$CH-), 42.81 (-<u>C</u>H$_2$Br), 53.73 (-Ph<u>C</u>H$_2$CH-), 56.33 (-O<u>C</u>H$_2$CCH), 76.09 (-OCH$_2$C<u>C</u>H), 77.96 (-OCH$_2$<u>C</u>CH), 115.74 (aromat.), 130.83 (aromat.), 157.48 (aromat.), 166.60 (-<u>C</u>OO-), 174.96 (-N<u>C</u>O-).
^{19}F-NMR ($CDCl_3$, 400 MHz) δ (ppm): -152.67 (d, 2F, *ortho*), -157.57 (t, 1F, *para*), -162.42 (t, 2F, *meta*).
Reactivity of the PFP ester precluded ESI-MS analysis.

1.2. Preparative synthesis of probe LS18

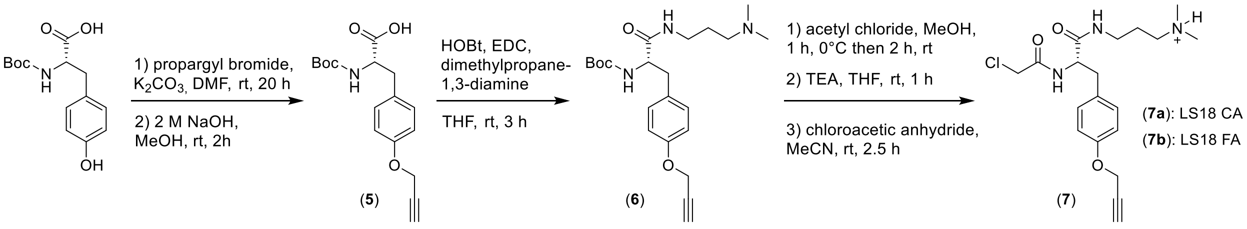

2-*tert*-Butoxycarbonylamino-3-[4-(prop-2-yn-1-yloxy)phenyl]propanoic acid (5)

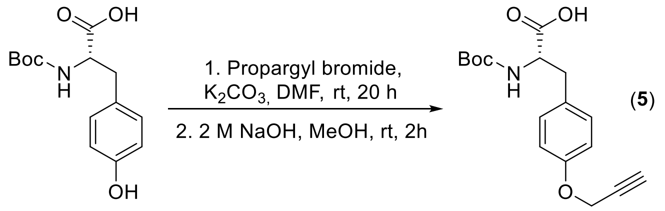

6.0 g Boc-L-tyrosine (21.3 mmol) and 9.0 g K_2CO_3 (65.2 mmol, 3 eq.) were dissolved in 30 mL dry DMF. 6.4 mL propargyl bromide (97%, 82.4 mmol, 3.9 eq.) was added and the reaction stirred at room temperature for 20h. Water was added and the mixture extracted with ether, washed with brine, dried with Na_2SO_4, filtered and the solvent evaporated. TLC showed one product with only traces of impurities (R_f = 0.38 (petrol ether/ethyl acetate 5:1)). The yellow oil was dissolved in 30 mL methanol and 42 mL 2 M NaOH solution was added. The mixture was stirred at room temperature for 2h, adjusted to pH = 4-5 with 3 M HCl solution and extracted with ethyl acetate. The organic phase was washed with brine, dried with Na_2SO_4, filtered and the solvent evaporated leaving the product (**5**) as yellow solid (m = 6.06 g, 89 %). The product was used in the next step without further purification.

^{1}H-NMR (MeOD, 600 MHz) δ (ppm): 1.38 (s, 9H, Boc), 2.86 (dd, 1H, *J* = 8.2 Hz, *J* = 13.9 Hz, H-3), 2.91 (t, 1H, *J* = 2.3 Hz, -C≡CH), 3.10 (dd, 1H, *J* = 5.0 Hz, *J* = 13.9 Hz, H-3), 4.24 (m, 1H, H-2), 4.68 (d, 2H, *J* = 2.3 Hz, -C**H**$_2$-C≡CH), 6.88 (d, 2H, *J* = 8.5 Hz, H-3´), 7.15 (d, 2H, *J* = 8.5 Hz, H-2´).
^{13}C-NMR (MeOD, 151 MHz) δ (ppm): 28.7 (3C, Boc), 38.3 (C-3), 56.6 (-**C**H$_2$-C≡CH), 57.4 (C-2), 76.6 (-CH$_2$-C≡**C**H), 80.0 (-CH$_2$-**C**≡CH), 80.3 (1C, Boc), 115.7 (C-3´), 131.4 (C-2´), 131.9 (C-1´), 157.7 (-NHCOO-), 157.9 (C-4´), 177.6 (C-1).
ESI-HRMS: m/z = 342.1312 $[M+Na]^+$, calc. for $C_{17}H_{21}NO_5 + Na^+$ = 342.1312.

***tert*-Butyl-(*S*)-{1-([3-(dimethylamino)propyl]amino)-1-oxo-3-[4-(prop-2-yn-1-yloxy)phenyl]propan-2-yl}carbamate (6)**

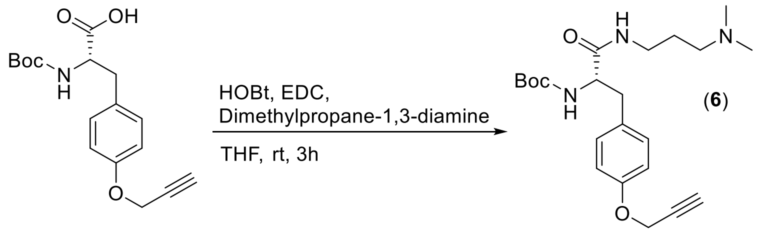

6.06 g (**5**) (18.975 mmol) was dissolved in 65 mL THF. 4.8 g HOBt (30.4 mmol, 1.6 eq.) and 4.42 g EDC HCl (22.8 mmol, 1.2 eq.) was added. After 5 min, 3.6 mL dimethylpropane-1,3-diamine (28.5 mmol, 1.5 eq.) was added. The reaction was stirred at room temperature for 3h. Water was added and the mixture extracted with ethyl acetate, washed with $NaHCO_3$ and brine, dried with Na_2SO_4, filtered and the solvent evaporated leaving the product (**5**) as yellow solid (m = 7.43 g, 97 %). The product was used in the next step without further purification.

S4_2: ^{1}H-NMR (MeOD 600.33 MHz) δ (ppm): 1.39 (s, 9H, Boc), 1.63 (m, 2H, -CH$_2$-C**H**$_2$-CH$_2$-N-(CH$_3$)$_2$), 2.30 (s, 6H, -N-(CH$_3$)$_2$), 2.37 (m, 2H, -CH$_2$-CH$_2$-C**H**$_2$-N-(CH$_3$)$_2$), 2.79 (dd, 1H, *J* = 8.1 Hz, *J* = 13.9 Hz, H-3), 2.93 (t, 1H, *J* = 2.2 Hz, -C≡CH), 2.98 (dd, 1H, *J* = 6.7 Hz, *J* = 13.9 Hz, H-3), 3.16 (m, 2H, -C**H**$_2$-CH$_2$-CH$_2$-N-(CH$_3$)$_2$), 4.18 (dd, 1H, *J* = 6.7 Hz, *J* = 8.1 Hz, H-2), 4.69 (d, 2H, *J* = 2.2 Hz, -C**H**$_2$-C≡CH), 6.91 (d, 2H, *J* = 8.2 Hz, H-3´), 7.16 (d, 2H, *J* = 8.2 Hz, H-2´).
^{13}C-NMR (MeOD 150.97 MHz) δ (ppm): 27.5 (-CH$_2$-**C**H$_2$-CH$_2$-N-(CH$_3$)$_2$), 28.7 (3C, Boc), 38.3 (-**C**H$_2$-CH$_2$-CH$_2$-N-(CH$_3$)$_2$), 38.5 (C-3), 45.1 (2C, -N-(CH$_3$)$_2$), 56.6 (-**C**H$_2$-C≡CH), 57.8 (2C, -CH$_2$-CH$_2$-**C**H$_2$-N-(CH$_3$)$_2$ and

C-2), 76.7 (-CH_2-C≡**C**H), 79.9 (-CH_2-**C**≡CH), 80.6 (1C, Boc), 115.9 (C-3´), 131.3 (C-2´ and C-1´), 157.5 (-NHCOO-), 158.0 (C-4´), 174.4 (C-1).
ESI-HRMS: m/z = 404.2532 $[M+H]^+$, calc. for $C_{22}H_{33}N_3O_4 + H^+$ = 404.2544.

(*S*)-2-amino-*N*-[3-(dimethylamino)propyl]-3-[4-(prop-2-yn-1-yloxy)phenyl]propanamide (6b)

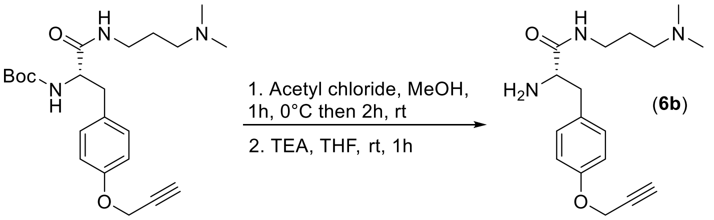

1.5 g (**6**) (3.717 mmol) was dissolved in 37 mL MeOH and 3.73 mL acetyl chloride (52 mmol, 14 eq.) was added at 0°C. The reaction was stirred at this temperature for 1 h and then 2 h at room temperature. The solvent was evaporated and the residue dried under high vacuum leaving a light yellow solid foam (quantitative yield) for the unprotected dihydrochloride. For desalting, 720 mg of the dihydrochloride (1.91 mmol) was dissolved in 20 mL THF and 585 µl TEA (4.22 mmol, 2.2 eq.) was added and the reaction stirred for 1 h at room temperature. The precipitate was filtered off and the filtrate reduced under vacuum leaving the product as yellow oil (m = 523 mg, 90%).

^{1}H-NMR (MeOD, 600 MHz) δ (ppm): 1.58 (m, 2H, -CH_2-C**H**$_2$-CH_2-N-$(CH_3)_2$), 2.25 (s, 6H, -N-$(CH_3)_2$), 2.27 (m, 2H, -CH_2-CH_2-C**H**$_2$-N-$(CH_3)_2$), 2.80 (dd, 1H, *J* = 6.7 Hz, *J* = 13.5 Hz, H-3), 2.88 (dd, 1H, *J* = 7.3 Hz, *J* = 13.5 Hz, H-3), 2.93 (t, 1H, *J* = 2.3 Hz, -C≡CH), 3.10 and 3.18 (m, 2H, -C**H**$_2$-CH_2-CH_2-N-$(CH_3)_2$), 3.47 (dd, 1H, *J* = 7.0 Hz, *J* = 7.8 Hz, H-2), 4.69 (s, 2H, -C**H**$_2$-C≡CH), 6.92 (d, 2H, *J* = 8.6 Hz, H-3´), 7.13 (d, 2H, *J* = 8.6 Hz, H-2´). ^{13}C-NMR (MeOD, 151 MHz) δ (ppm): 27.8 (-CH_2-**C**H_2-CH_2-N-$(CH_3)_2$), 38.3 (-**C**H_2-CH_2-CH_2-N-$(CH_3)_2$), 41.7 (C-3), 45.2 (2C, -N-$(CH_3)_2$), 56.6 (-**C**H_2-C≡CH), 57.9 (2C, -CH_2-CH_2-**C**H_2-N-$(CH_3)_2$ and C-2), 76.7 (-CH_2-C≡**C**H), 79.9 (-CH_2-**C**≡CH), 116.0 (C-3´), 131.4 (C-2´), 131.5 (C-1´), 158.1 (C-4´), 176.5 (C-1).
ESI-HRMS: m/z = 304.2011 $[M+H]^+$, calc. for $C_{17}H_{25}N_3O_2 + H^+$ = 304.2020.

(*S*)-3-{2-(2-Chloroacetamido)-3-[4-(prop-2-yn-1-yloxy)phenyl]propanamido}-*N*,*N*-dimethylpropan-1-aminium salts (7a and 7b)

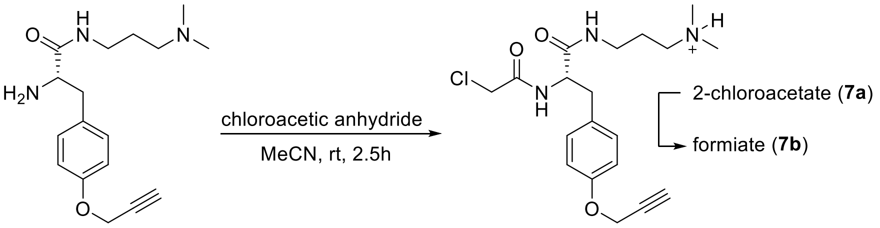

96 mg desalted amine (**6b**) (0.316 mmol) was dissolved in 5 mL dry MeCN and 68.4 mg chloroacetic anhydride (0.38 mmol, 1.2 eq.) was added. The reaction was stirred for 2.5 h at room temperature. The reaction was quenched by the addition of 50 µl water and stirred for another 5 min. The solvent and volatile components were evaporated under vacuum. The compound was purified by column chromatography on silica gel with pure methanol. The compound was received as chloroacetate salt (**7a**) as colorless oil (m = 123 mg, 82%). R_f = 1.6 (MeOH). For biological experiments the compound was further purified by prep. HPLC (A: H_2O + 0.1 % FA, B: MeOH + 0.1 % FA; 5% B to 95% B) to give the clean compound as formate (**7b**) salt as colorless oil.

LS18 CA (**7a**): ^{1}H-NMR (MeOD 400 MHz) δ (ppm): 1.83 (m, 2H, -CH_2-C**H**$_2$-CH_2-N-$(CH_3)_2$), 2.83 (s, 6H, -N-$(CH_3)_2$), 2.91 – 3.07 (m, 5H, -C≡CH, -CH_2-CH_2-C**H**$_2$-N-$(CH_3)_2$, H-3), 3.17 and 3.26 (m, 2H, -C**H**$_2$-CH_2-CH_2-N-

$(CH_3)_2$), 3.99 (s, 2H, chloroacetate), 4.06 (s, 2H, -CH_2-Cl), 4.42 (dd, 1H, J = 7.5 Hz, *J* = 7.9 Hz, H-2), 4.70 (s, 2H, -C**H**$_2$-C≡CH), 6.93 (d, 2H, *J* = 8.2 Hz, H-3´), 7.18 (d, 2H, *J* = 8.3 Hz, H-2´).
^{13}C-NMR (MeOD 101 MHz) δ (ppm): 25.8 (-CH_2-**C**H_2-CH_2-N-$(CH_3)_2$), 36.8 (-**C**H_2-CH_2-CH_2-N-$(CH_3)_2$), 37.7 (C-3), 43.0 (-CH_2-Cl), 43.5 (2C, -N-$(CH_3)_2$), 44.4 (-CH_2-Cl chloroacetate), 56.4 (-CH_2-CH_2-**C**H_2-N-$(CH_3)_2$), 56.7 (-**C**H_2-C≡CH), 57.2 (C-2), 76.7 (-CH_2-C≡**C**H), 79.9 (-CH_2-**C**≡CH), 116.1 (C-3´), 130.7 (C-1´), 131.3 (C-2´), 158.2 (C-4´), 169.2 (-**C**O-CH_2-Cl), 173.8 (-COO^- chloroacetate), 174.0 (C-1).

LS18 FA (**7b**): ^{1}H-NMR (MeOD 400 MHz) δ (ppm): 1.84 (m, 2H, -CH_2-C**H**$_2$-CH_2-N-$(CH_3)_2$), 2.82 (s, 6H, -N-$(CH_3)_2$), 2.89 – 3.09 (m, 5H, -C≡CH, -CH_2-CH_2-C**H**$_2$-N-$(CH_3)_2$, H-3), 3.18 and 3.26 (m, 2H, -C**H**$_2$-CH_2-CH_2-N-$(CH_3)_2$), 4.06 (s, 2H, -CH_2-Cl), 4.43 (dd, 1H, *J* = 7.5 Hz, *J* = 8.0 Hz, H-2), 4.70 (s, 2H, -C**H**$_2$-C≡CH), 6.93 (d, 2H, *J* = 8.4 Hz, H-3´), 7.18 (d, 2H, *J* = 8.4 Hz, H-2´), 8.42 (s, br, 1H, formate ion).
^{13}C-NMR (MeOD 101 MHz) δ (ppm): 25.7 (-CH_2-**C**H_2-CH_2-N-$(CH_3)_2$), 36.8 (-**C**H_2-CH_2-CH_2-N-$(CH_3)_2$), 37.7 (C-3), 43.0 (-CH_2-Cl), 43.4 (2C, -N-$(CH_3)_2$), 56.3 (-CH_2-CH_2-**C**H_2-N-$(CH_3)_2$), 56.6 (-**C**H_2-C≡CH), 57.2 (C-2), 76.8 (-CH_2-C≡**C**H), 79.9 (-CH_2-**C**≡CH), 116.1 (C-3´), 130.7 (C-1´), 131.3 (C-2´), 158.2 (C-4´), 168.4 (formate ion), 169.1 (-**C**O-CH_2-Cl), 173.9 (C-1).
ESI-HRMS: m/z = 380.1737 $[M]^+$, calc. for $C_{19}H_{27}ClN_3O_3^+$ = 380.1735.

2. Recombinant proteins

2.1. Plasmid preparation

The gene coding for the herein used proteins (t3CL^{pro}, PL^{pro} and their mutants) were custom synthesized and constructed in a pET-51b(+) plasmid (GenScript, New Jersey) for protein expression in *E. coli* BL21 cells. The expression vector was IPTG inducible with ampicillin marker and *N*-terminal Strep-tag II with cloning site KpnI-BamHI.

Sequences of the genes and proteins:

SARS-CoV-2

t3CL^{pro}

GGTACCGAGCGGTTTCCGTAAGATGGCGTTTCCGAGCGGCAAAGTTGAGGGTTGCATGGTGCAGGTTACCTG
CGGCACCACCACCCTGAACGGTCTGTGGCTGGACGATGTGGTTTACTGCCCGCGTCACGTTATCTGCACCAGC
GAGGACATGCTGAACCCGAACTATGAAGATCTGCTGATTCGTAAGAGCAACCACAACTTCCTGGTGCAGGCGG
GCAACGTGCAACTGCGTGTTATCGGTCACAGCATGCAGAACTGCGTGCTGAAGCTGAAAGTTGACACCGCGA
ACCCGAAAACCCCGAAGTACAAATTCGTGCGTATTCAGCCGGGCCAAACCTTTAGCGTTCTGGCGTGCTACAA
CGGCAGCCCGAGCGGTGTTTATCAATGCGCGATGCGTCCGAACTTCACCATCAAGGGTAGCTTTCTGAACGGT
AGCTGCGGCAGCGTGGGTTTCAACATTGACTACGATTGCGTTAGCTTTTGCTATATGCACCACATGGAGCTGCC
GACCGGCGTTCATGCGGGCACCGATCTGGAAGGCAACTTCTACGGTCCGTTTGTTGACCGTCAAACCGCGCAA
GCGGCGGGCACCGATACCACCATCACCGTGAACGTTCTGGCGTGGCTGTATGCGGCGGTGATTAACGGTGAC
CGTTGGTTCCTGAACCGTTTTACCACCACCCTGAACGATTTCAACCTGGTGGCGATGAAGTACAACTATGAGCC
GCTGACCCAGGACCACGTTGATATCCTGGGCCCGCTGAGCGCGCAAACCGGTATTGCGGTGCTGGACATGTGC
GCGAGCCTGAAAGAACTGCTGCAAAACGGCATGAACGGTCGTACCATTCTGGGCAGCGCGCTGCTGGAGGAC
GAATTCACCCCGTTTGATGTGGTTCGTCAGTGCAGCGGTGTGACCTTTCAATAAGGATCC

Protein sequence:

SGFRKMAFPSGKVEGCMVQVTCGTTTLNGLWLDDVVYCPRHVICTSEDMLNPNYEDLLIRKSNHNFLVQAGNVQ
LRVIGHSMQNCVLKLKVDTANPKTPKYKFVRIQPGQTFSVLACYNGSPSGVYQCAMRPNFTIKGSFLNGS**C**GSVGF
NIDYDCVSFCYMHHMELPTGVHAGTDLEGNFYGPFVDRQTAQAAGTDTTITVNVLAWLYAAVINGDRWFLNRFT

TTLNDFNLVAMKYNYEPLTQDHVDILGPLSAQTGIAVLDMCASLKELLQNGMNGRTILGSALLEDEFTPFDVVRQC
SGVTFQ

t3CL^{pro} mutant (C145 to A145)

GGTACCGAGCGGTTTCCGTAAGATGGCGTTTCCGAGCGGCAAAGTTGAGGGTTGCATGGTGCAGGTTACCTG
CGGCACCACCACCCTGAACGGTCTGTGGCTGGACGATGTGGTTTACTGCCCGCGTCACGTTATCTGCACCAGC
GAGGACATGCTGAACCCGAACTATGAAGATCTGCTGATTCGTAAGAGCAACCACAACTTCCTGGTGCAGGCGG
GCAACGTGCAACTGCGTGTTATCGGTCACAGCATGCAGAACTGCGTGCTGAAGCTGAAAGTTGACACCGCGA
ACCCGAAAACCCCGAAGTACAAATTCGTGCGTATTCAGCCGGGCCAAACCTTTAGCGTTCTGGCGTGCTACAA
CGGCAGCCCGAGCGGTGTTTATCAATGCGCGATGCGTCCGAACTTCACCATCAAGGGTAGCTTTCTGAACGGT
AGCgcaGGCAGCGTGGGTTTCAACATTGACTACGATTGCGTTAGCTTTTGCTATATGCACCACATGGAGCTGCC
GACCGGCGTTCATGCGGGCACCGATCTGGAAGGCAACTTCTACGGTCCGTTTGTTGACCGTCAAACCGCGCAA
GCGGCGGGCACCGATACCACCATCACCGTGAACGTTCTGGCGTGGCTGTATGCGGCGGTGATTAACGGTGAC
CGTTGGTTCCTGAACCGTTTTACCACCACCCTGAACGATTTCAACCTGGTGGCGATGAAGTACAACTATGAGCC
GCTGACCCAGGACCACGTTGATATCCTGGGCCCGCTGAGCGCGCAAACCGGTATTGCGGTGCTGGACATGTGC
GCGAGCCTGAAAGAACTGCTGCAAAACGGCATGAACGGTCGTACCATTCTGGGCAGCGCGCTGCTGGAGGAC
GAATTCACCCCGTTTGATGTGGTTCGTCAGTGCAGCGGTGTGACCTTTCAATAAGGATCC

Protein sequence:

SGFRKMAFPSGKVEGCMVQVTCGTTTLNGLWLDDVVYCPRHVICTSEDMLNPNYEDLLIRKSNHNFLVQAGNVQ
LRVIGHSMQNCVLKLKVDTANPKTPKYKFVRIQPGQTFSVLACYNGSPSGVYQCAMRPNFTIKGSFLNGS**A**GSVGF
NIDYDCVSFCYMHHMELPTGVHAGTDLEGNFYGPFVDRQTAQAAGTDTTITVNVLAWLYAAVINGDRWFLNRFT
TTLNDFNLVAMKYNYEPLTQDHVDILGPLSAQTGIAVLDMCASLKELLQNGMNGRTILGSALLEDEFTPFDVVRQC
SGVTFQ

PL^{pro}

GGTACCGAGCCTGCGTGAGGTTCGTACCATCAAAGTTTTTACCACCGTGGATAACATCAATCTGCACACCCAAG
TTGTGGATATGAGCATGACCTACGGTCAGCAATTCGGCCCGACCTATCTGGACGGCGCGGATGTGACCAAGAT
CAAACCGCACAACAGCCACGAGGGCAAGACCTTTTACGTGCTGCCGAACGACGATACCCTGCGTGTTGAGGCG
TTCGAATACTATCACACCACCGACCCGAGCTTTCTGGGCCGTTACATGAGCGCGCTGAACCACACCAAGAAATG
GAAGTATCCGCAAGTTAACGGTCTGACCAGCATCAAATGGGCGGACAACAACTGCTATCTGGCGACCGCGCTG
CTGACCCTGCAGCAAATTGAGCTGAAGTTCAACCCGCCGGCGCTGCAGGATGCGTACTATCGTGCGCGTGCGG
GCGAAGCGGCGAACTTTTGCGCGCTGATCCTGGCGTACTGCAACAAAACCGTGGGCGAGCTGGGCGACGTTC
GTGAAACCATGAGCTACCTGTTCCAACACGCGAACCTGGATAGCTGCAAGCGTGTGCTGAACGTGGTTTGCAA
AACCTGCGGTCAGCAACAGACCACCCTGAAGGGCGTGGAGGCGGTTATGTACATGGGCACCCTGAGCTATGA
ACAATTTAAGAAAGGCGTTCAGATTCCGTGCACCTGCGGCAAGCAGGCGACCAAATACCTGGTGCAACAGGA
AAGCCCGTTCGTTATGATGAGCGCTCCGCCGGCGCAATATGAGCTGAAACACGGCACCTTTACCTGCGCGAGC
GAATACACCGGTAACTATCAGTGCGGCCACTACAAGCACATCACCAGCAAAGAAACCCTGTATTGCATTGACG
GCGCGCTGCTGACCAAGAGCAGCGAATACAAAGGTCCGATTACCGATGTGTTTTACAAAGAGAACAGCTACAC
CACCACCATCAAGCCGGTTACCTACAAACTGGACGGTTAAGGATCC

Protein sequence:

SLREVRTIKVFTTVDNINLHTQVVDMSMTYGQQFGPTYLDGADVTKIKPHNSHEGKTFYVLPNDDTLRVEAFEYYH
TTDPSFLGRYMSALNHTKKWKYPQVNGLTSIKWADNN**C**YLATALLTLQQIELKFNPPALQDAYYRARAGEAANFCA
LILAYCNKTVGELGDVRETMSYLFQHANLDSCKRVLNVVCKTCGQQQTTLKGVEAVMYMGTLSYEQFKKGVQIPC
TCGKQATKYLVQQESPFVMMSAPPAQYELKHGTFTCASEYTGNYQCGHYKHITSK
ETLYCIDGALLTKSSEYKGPITDVFYKENSYTTTIKPVTYKLDG

PL^{pro} mutant (C114 to A114)

GGTACCGAGCCTGCGTGAGGTTCGTACCATCAAAGTTTTTACCACCGTGGATAACATCAATCTGCACACCCAAG
TTGTGGATATGAGCATGACCTACGGTCAGCAATTCGGCCCGACCTATCTGGACGGCGCGGATGTGACCAAGAT
CAAACCGCACAACAGCCACGAGGGCAAGACCTTTTACGTGCTGCCGAACGACGATACCCTGCGTGTTGAGGCG
TTCGAATACTATCACACCACCGACCCGAGCTTTCTGGGCCGTTACATGAGCGCGCTGAACCACACCAAGAAATG
GAAGTATCCGCAAGTTAACGGTCTGACCAGCATCAAATGGGCGGACAACAACgctTATCTGGCGACCGCGCTG
CTGACCCTGCAGCAAATTGAGCTGAAGTTCAACCCGCCGGCGCTGCAGGATGCGTACTATCGTGCGCGTGCGG
GCGAAGCGGCGAACTTTTGCGCGCTGATCCTGGCGTACTGCAACAAAACCGTGGGCGAGCTGGGCGACGTTC
GTGAAACCATGAGCTACCTGTTCCAACACGCGAACCTGGATAGCTGCAAGCGTGTGCTGAACGTGGTTTGCAA
AACCTGCGGTCAGCAACAGACCACCCTGAAGGGCGTGGAGGCGGTTATGTACATGGGCACCCTGAGCTATGA
ACAATTTAAGAAAGGCGTTCAGATTCCGTGCACCTGCGGCAAGCAGGCGACCAAATACCTGGTGCAACAGGA
AAGCCCGTTCGTTATGATGAGCGCTCCGCCGGCGCAATATGAGCTGAAACACGGCACCTTTACCTGCGCGAGC
GAATACACCGGTAACTATCAGTGCGGCCACTACAAGCACATCACCAGCAAAGAAACCCTGTATTGCATTGACG
GCGCGCTGCTGACCAAGAGCAGCGAATACAAAGGTCCGATTACCGATGTGTTTTACAAAGAGAACAGCTACAC
CACCACCATCAAGCCGGTTACCTACAAACTGGACGGTTAAGGATCC

Protein sequence:

SLREVRTIKVFTTVDNINLHTQVVDMSMTYGQQFGPTYLDGADVTKIKPHNSHEGKTFYVLPNDDTLRVEAFEYYH
TTDPSFLGRYMSALNHTKKWKYPQVNGLTSIKWADNNAYLATALLTLQQIELKFNPPALQDAYYRARAGEAANFC
ALILAYCNKTVGELGDVRETMSYLFQHANLDSCKRVLNVVCKTCGQQQTTLKGVEAVMYMGTLSYEQFKKGVQIP
CTCGKQATKYLVQQESPFVMMSAPPAQYELKHGTFTCASEYTGNYQCGHYKHITSK
ETLYCIDGALLTKSSEYKGPITDVFYKENSYTTTIKPVTYKLDG

SARS-CoV-1

t3CL^{pro}

GGTACCGAGCGGTTTCCGTAAGATGGCGTTTCCGAGCGGCAAAGTTGAGGGTTGCATGGTGCAGGTTACCTG
CGGCACCACCACCCTGAACGGTCTGTGGCTGGACGATACCGTGTACTGCCCGCGTCACGTTATCTGCACCGCG
GAGGACATGCTGAACCCGAACTATGAAGATCTGCTGATTCGTAAGAGCAACCACAGCTTCCTGGTTCAGGCGG
GCAACGTGCAACTGCGTGTTATCGGTCACAGCATGCAGAACTGCCTGCTGCGTCTGAAGGTGGACACCAGCAA
CCCGAAAACCCCGAAGTACAAATTCGTGCGTATTCAGCCGGGCCAAACCTTTAGCGTTCTGGCGTGCTACAAC
GGCAGCCCGAGCGGTGTGTATCAATGCGCGATGCGTCCGAACCACACCATCAAGGGTAGCTTTCTGAACGGTA
GCTGCGGCAGCGTGGGTTTCAACATTGACTACGATTGCGTTAGCTTTTGCTATATGCACCACATGGAGCTGCCG
ACCGGCGTTCATGCGGGCACCGATCTGGAAGGCAAATTCTACGGTCCGTTTGTTGACCGTCAAACCGCGCAAG
CGGCGGGCACCGATACCACCATCACCCTGAACGTGCTGGCGTGGCTGTATGCGGCGGTTATTAACGGTGACCG
TTGGTTCCTGAACCGTTTTACCACCACCCTGAACGATTTCAACCTGGTGGCGATGAAGTACAACTATGAGCCGC
TGACCCAGGACCACGTTGATATCCTGGGCCCGCTGAGCGCGCAAACCGGTATTGCGGTGCTGGACATGTGCGC
GGCGCTGAAAGAACTGCTGCAAAACGGCATGAACGGTCGTACCATCCTGGGCAGCACCATTCTGGAGGACGA
ATTCACCCCGTTTGATGTGGTTCGTCAGTGCAGCGGTGTTACCTTTCAATAAGGATCC

Protein sequence:

SGFRKMAFPSGKVEGCMVQVTCGTTTLNGLWLDDTVYCPRHVICTAEDMLNPNYEDLLIRKSNHSFLVQAGNVQL
RVIGHSMQNCLLRLKVDTSNPKTPKYKFVRIQPGQTFSVLACYNGSPSGVYQCAMRPNHTIKGSFLNGSCGSVGFN
IDYDCVSFCYMHHMELPTGVHAGTDLEGKFYGPFVDRQTAQAAGTDTTITLNVLAWLYAAVINGDRWFLNRFTTT
LNDFNLVAMKYNYEPLTQDHVDILGPLSAQTGIAVLDMCAALKELLQNGMNGRT
ILGSTILEDEFTPFDVVRQCSGVTFQ

t3CL^{pro} mutant (C145 to A145)

GGTACCGAGCGGTTTCCGTAAGATGGCGTTTCCGAGCGGCAAAGTTGAGGGTTGCATGGTGCAGGTTACCTG
CGGCACCACCACCCTGAACGGTCTGTGGCTGGACGATACCGTGTACTGCCCGCGTCACGTTATCTGCACCGCG
GAGGACATGCTGAACCCGAACTATGAAGATCTGCTGATTCGTAAGAGCAACCACAGCTTCCTGGTTCAGGCGG
GCAACGTGCAACTGCGTGTTATCGGTCACAGCATGCAGAACTGCCTGCTGCGTCTGAAGGTGGACACCAGCAA
CCCGAAAACCCCGAAGTACAAATTCGTGCGTATTCAGCCGGGCCAAACCTTTAGCGTTCTGGCGTGCTACAAC
GGCAGCCCGAGCGGTGTGTATCAATGCGCGATGCGTCCGAACCACACCATCAAGGGTAGCTTTCTGAACGGTA
GCgcaGGCAGCGTGGGTTTCAACATTGACTACGATTGCGTTAGCTTTTGCTATATGCACCACATGGAGCTGCCG
ACCGGCGTTCATGCGGGCACCGATCTGGAAGGCAAATTCTACGGTCCGTTTGTTGACCGTCAAACCGCGCAAG
CGGCGGGCACCGATACCACCATCACCCTGAACGTGCTGGCGTGGCTGTATGCGGCGGTTATTAACGGTGACCG
TTGGTTCCTGAACCGTTTTACCACCACCCTGAACGATTTCAACCTGGTGGCGATGAAGTACAACTATGAGCCGC
TGACCCAGGACCACGTTGATATCCTGGGCCCGCTGAGCGCGCAAACCGGTATTGCGGTGCTGGACATGTGCGC
GGCGCTGAAAGAACTGCTGCAAAACGGCATGAACGGTCGTACCATCCTGGGCAGCACCATTCTGGAGGACGA
ATTCACCCCGTTTGATGTGGTTCGTCAGTGCAGCGGTGTTACCTTTCAATAAGGATCC

Protein sequence:

SGFRKMAFPSGKVEGCMVQVTCGTTTLNGLWLDDTVYCPRHVICTAEDMLNPNYEDLLIRKSNHSFLVQAGNVQL
RVIGHSMQNCLLRLKVDTSNPKTPKYKFVRIQPGQTFSVLACYNGSPSGVYQCAMRPNHTIKGSFLNGS**A**GSVGFN
IDYDCVSFCYMHHMELPTGVHAGTDLEGKFYGPFVDRQTAQAAGTDTTITLNVLAWLYAAVINGDRWFLNRFTTT
LNDFNLVAMKYNYEPLTQDHVDILGPLSAQTGIAVLDMCAALKELLQNGMNGRT
ILGSTILEDEFTPFDVVRQCSGVTFQ

By cloning into the expression vector each of the resulting protein sequences additionally featured the *N*-terminal Strep-tag II with the enterokinase cleavage site: MASWSHPQFEKGADDDDKVP followed by the above listed native protein sequences.

2.2. Transformation of plasmids

For preparation of competent cells, 1 mL of an overnight culture of *E. coli* BL21 cells was inoculated in 25 mL of LB medium and kept at 37 °C and 180 rpm. At an OD_{600} of 0.5 the cells were transferred into a 50 mL tarson tube and kept on ice for 20 min. Centrifugation at 6000 rpm and 4 °C for 10 min was performed. The supernatant was discarded and the cell pellet resuspended into 20 mL ice cold calcium chloride (50 mM). After an incubation time of 20 min on ice the cells were centrifuged at 6000 rpm for 10 min at 4 °C and the supernatant discarded. The cell pellet was again resuspended in 4.25 mL of calcium chloride solution (50 mM) as well as 0.75 mL of glycerol. Aliquots of 200 µL were made and directly put into liquid nitrogen before storing them at -80 °C.

An aliquot of competent *E. coli* BL21 cells was thawed on ice. 1 µL of the corresponding plasmid (50 ng/mL) was added to the cells and mixed by tabbing the tube three times. After incubation on ice for 30 min the cells were heat shocked at 42 °C for 45 sec. Afterwards the tube was placed on ice for 3 min. 900 µL LB media was added to the cells and they were kept at 37 °C for 1 h whilst shaking at 550 rpm. Centrifugation at 8000 rpm and 4 °C for 2 min was performed. The supernatant was discarded and the cell pellet resuspended. The cell suspension was streaked on ampicillin plates (100 µg/L), which were kept at 37 °C overnight. The next day colonies could be picked for overnight cultures which could be used to prepare glycerol stocks.

2.3. Overproduction of proteins

Overnight cultures for cellular assays were prepared by taking a small amount of a bacterial cryo-stock (15 % glycerol, stored at -80 °C) and inoculating them in 5 mL LB in sterile 13 mL polypropylene tubes (Sarstedt, ref 62.515.028), supplemented with antibiotics as indicated and grown for 14-16 h at 37 °C (180 rpm). 1 mL of a corresponding overnight culture was inoculated in 50 mL LB medium containing 100 µL/mL ampicillin and kept at 37 °C and 180 rpm. At an OD_{600} of 0.3 the protein expression was induced by adding 0.2 µg/mL of an IPTG solution (1 M). The cells were incubated at 37 °C and 180 rpm for 2 h before centrifugation at 4000 rpm for 20 min at 4 °C.

2.4. Affinity-purification of proteins

For purifying the proteins overexpression was performed in a scale of 3 L as described. The cell culture was centrifuged down at 4000 rpm for 20 min at 4 °C. The supernatant was discarded and the cell pellet washed with 15 mL PBS before centrifuging at 4000 rpm for 20 min at 4 °C. The cells were lysed by ultrasound treatment (25 % amplitude, 0.5 s ON, 2.1 s OFF, 20 pulses, Branson Digital Sonifier). Afterwards, samples were centrifuged for 1 h at 4000 rpm and 4 °C. The supernatant was transferred into a new 50 mL flask and put on ice before performing affinity purification via Strep-tag II on an ÄKTA start (GE Healthcare) using StrepTrap HP columns (GE Healthcare). Standard Bradford protocols were used to calculate the concentration of the protein fractions. 200 µL Aliquots of 0.4 mg/mL protein were frozen in liquid nitrogen before storing them at -80 °C.

2.5. Enterokinase digest of t3CL^{pro}

For cleavage of the *N*-terminal Strep-tag II, 2 µL Enterokinase (0.3 mg/mL, Boehringer Mannheim) was added to 25 µL purified recombinant t3CL^{pro} (0.8 mg/mL) in phosphate-free buffer (50 mM Tris-HCl, 1 mM EDTA, pH = 7.3) and the mixture was incubated at 37 °C for 3.5 h.

3. Probe reactions

3.1. LS-probe modification with ligands

In a 1.5 mL micro reaction tube were added 2.5 µL of 5 M pyridine (in DMSO), 2.5 µL of a probe stock (1 mM in butyl acetate) as well as 2.5 µL of the corresponding amine containing ligand (1 mM in DMSO) to result in a final concentration of 50 µM in the cell suspension. The mixture was incubated for 1 h at room temperature. Quenching was performed by adding 50 µL butyl acetate to the reaction mixture and pipetting the entire solution into a fresh 1.5 mL micro reaction tube containing 5 mg of (aminomethyl)polystyrene beads (70-90 mesh, Sigma-Aldrich). After incubation for 15 min the supernatant was transferred into a fresh 1.5 mL micro reaction tube. The beads were washed with 50 µL of butyl acetate and the supernatants were combined. The pooled solution was dried at high vacuum. 2 µL of DMSO were used to dissolve the reacted probe. For dose down experiments the same procedure as for the reaction of a LS-probe and ligand was used. To get a dose down of a final concentration of 50 µM, 20 µM, 10 µM, 5 µM, 1 µM and 0.1 µM in 50 µL the respective amount of ligand and probe was used.

3.2. *In situ* probe labelling of proteins in live *E. coli* cells

After overexpressing a protein in *E. coli*, for each sample 1 mL of the induced bacterial culture was transferred into a 1.5 mL Eppendorf tube. The cells were pelleted by centrifugation (4000 rpm, 7 min, 4 °C), washed with 50 µL PBS before re-suspending them in 48 µL PBS. The reacted and quenched probes, dissolved in 2 µL DMSO were added to the cell suspension before incubation at 400 rpm at 37 °C for 1 h. After incubation the cells were pelleted by centrifugation (4000 rpm, 5 min, 4 °C) washed with 50 µL PBS and resuspended in 50 µL PBS. The cell suspension was stored at -80 °C before further

processing. After thawing the samples, they were lysed by ultrasound treatment (10 % amplitude, 0.5 s ON, 1 s OFF, 10 pulses, Branson Digital Sonifier). The resulting lysates were used for click chemistry and SDS-PAGE. After fluorescence scanning, Coomassie staining was applied to compare protein concentrations in the gel and validate the experiments.

3.3. *In vitro* probe labelling of proteins in lysates and purified protein

The respective protein aliquot (0.4 mg/mL) was thawed on ice. Reaction of LS probe and ligands was done as described. The reacted probe was dissolved in 2 µL DMSO and added with 8 µL PBS to 10 µL protein solution before incubation for 30 min at 400 rpm and 37 °C. After incubation Click Chemistry was performed.

3.4. LS-probe labelling of virus proteases in background of A549 and HepG2 proteomes

A549 human adenocarcinoma lung epithelial cells (ATCC, CCL-185) and human hepatocellular carcinoma HepG2 (ATCC, HB-8065) were maintained in DMEM (4.5 g/l glucose, pyruvate) (Gibco), supplemented with 10 % fetal bovine serum (PAA Laboratories) and penicillin/streptomycin (25 µg/ml each) at 37 °C, 5 % CO_2 (Gibco). HepG2 cells were seeded in T75 flasks at a density of 60.000 cells/cm^2 and allowed to proliferate for 3 days. A549 cells were seeded in T175 flasks at a density of 80.000 cells/cm^2 and allowed to proliferate for 4 days. For the experiments, 2 x 10^8 cells (HepG2) and 5 x 10^8 cells (A549) were harvested and lysed.

The cell pellets were resuspended in 10 mL PBS buffer and lysed *via* sonification using a Branson Digital Sonifier (25 % amplitude, 0.5 s ON, 2.1 s OFF, 20 pulses). After centrifugation for 20 min at 9000 rpm and 4 °C, the supernatant was frozen immediately in liquid N_2 and stored in aliquots at - 80 °C.

For the labelling assay, the cell lysate was thawed on ice and diluted with PBS to a protein concentration of 1.5 mg/mL in 38 µL. 2 µL of freshly synthesized LS probe (Stock 0.5 mM in DMSO, final concentration in the assay of 20 µM) and 10 µL of the protein (n3CL^{pro}, t3CL^{pro} or PL^{pro}) diluted in PBS was added to give the desired concentrations in a total volume of 50 µL. The final concentration of proteomes was 1.14 mg/mL based on 50 µL. The final concentrations of n3CL^{pro} and t3CL^{pro} and percentages of 3CL^{pro} in the total proteome for A549 cells were 200 µg/mL (15%), 100 µg/mL (8%), 20 µg/mL (1.7%), 10 µg/mL (0.9%), 2 µg/mL (0.18%), 1 µg/mL (0.09%), 0.5 µg/mL (0.04%), 0.1 µg/mL (0.009%). After incubation at 37 °C, 400 rpm for 30 min, click chemistry and SDS-PAGE was performed as described.

3.5. Competitive profiling experiments

3.5.1. *In situ* competitive profiling

The respective protein was overproduced in *E. coli* BL21 cells as described before. Reaction of LS probe and ligand was performed as described with 0.25 µL of LS-probe (1 mM), ligand (1 mM) and 5 M pyridine per sample. In the meantime, 1 µL of competitive molecule (10 mM) was incubated with 10 µL of the protein aliquot and 49 µL cell suspension for 1h at 25 °C and 400 rpm. Each sample of the reacted probe was dissolved in 0.25 µL DMSO and added to the cell suspension before incubation for 1 h at 400 rpm and 37 °C. The cells were further treated like described for *in situ* labeling with LS-probes.

3.5.2. In vitro competitive profiling

The respective protein aliquot (0.4 mg/mL) was thawed on ice. 10 µL of protein were used per reaction and pipetted into a 1.5 mL micro reaction tube together with 9.6 µL of PBS as well as 0.4 µL of the competitive compound (10 mM). The proteins were incubated for 30 min at 25 °C and 400 rpm. Reaction of probe and ligand was done as described with 0.1 µL LS-probe (1 mM), ligand (1mM) and pyridine (5 M) per sample. The reacted probe was dissolved in 0.1 µL DMSO and pipetted to the

proteins and incubated for 30 min at 37 °C and 400 rpm. Afterwards Click Chemistry was performed directly.

For dose down experiments the same procedure as for in vitro competitive profiling with LS-probes was performed. Final concentrations of 200 µM, 100 µM, 50 µM, 25 µM, 10 µM, 5 µM and 1 µM of the competitive compound were used with 5 µM of the reacted probe.

3.6. Click reactions

Click chemistry was performed using 40 µL cell lysate, 2 µL of a 0,65 mM rhodamine azide stock in DMSO, 4 µL of a 1.66 µM TBTA (tris((1-benzyl-4-triazolyl)methyl)amine) stock in *tert*-butanol/DMSO (8:2 v/v). To start the cycloaddition 2 µL of a freshly prepared 52 mM TCEP (tris(2-carboxyethyl)phosphine hydrochloride) stock in water and 2 µL of a 50 mM $CuSO_4$ stock solution in water were added. The samples were incubated for 1 h at room temperature before quenching with 50 µL 2x SDS loading buffer (63 mM Tris-HCl, 10 % (v/v) glycerol, 2 % (w/v) SDS, 0.0025 % (w/v) bromophenol blue, 10 % (v/v) β-mercaptoethanol; dissolved in water).

3.7. SDS-PAGE and in-gel fluorescence scanning

Before performing SDS-PAGE the samples were incubated for 10 min at 95 °C and subsequently centrifuged down. SDS gels containing 10 % acrylamide and an aqueous solution of 37.5:1 acrylamide and *N*,*N'*-methylenbisacrylamide were used with a PeqLab system and run at 75 mA per gel. Visualization was done by in-gel fluorescence scanning (Fusion-FX7). Equal protein content and separation in SDS-gels was confirmed by Coomassie staining (InstantBlue^{TM}, expedeon).

4. Proteomic experiments

In situ labelling was performed without doing click reaction subsequently. 2x SDS loading buffer was added directly to the resulting lysates. After SDS-PAGE and Coomassie staining the appropriate band was cut out of the gel and stored in a 1.5 mL micro reaction tube at 4 °C. Samples have been desalted using Nanosep 3K OMEGA centrifugal devices (Pall), digested with pepsin and measured on a Thermo LTQ Orbitrap Discovery with Eksigent 2D-nano HPLC (coverage of 84.4 %).

5. Substrate-based enzyme inhibition assays

All enzyme inhibition assays were performed in triplicates in black, flat-bottom 96-well plates with a total volume of 100 µL.

PL^{pro}: Purified recombinant enzyme was diluted to a concentration of 200 nM in PBS and 5 µL inhibitor dissolved in DMSO were added to the desired concentrations (250 µM, 100 µM, 50 µM, 25 µM, 10 µM, 2.5 µM, 1 µM, 0.5 µM). A positive control with 5 µL DMSO and a negative control without enzyme were performed alongside every measurement. After incubation for 10 min at room temperature, 5 µL of 2.5 mM fluorogenic substrate in DMSO (Arg-Leu-Arg-Gly-Gly-AMC, >98 % purity, Thermo Fisher) were added and fluorescence (λ_{ex} = 360 nm, λ_{em} = 460 nm) was measured at 37 °C every 30 s over 15 min in a Tecan M200 infinite Pro plate reader with 10 s of vigorous shaking before each measurement.

3CL^{pro}: Active n3CL^{pro} (*Sigma-Aldrich*) was diluted to a concentration of 200 nM in Tris-buffer (50 mM Tris-HCl, 1 mM EDTA, pH = 7.3) and 5 µL of inhibitor dissolved in DMSO were added to the desired concentrations (250 µM, 100 µM, 50 µM, 25 µM, 10 µM, 5 µM, 2.5 µM, 1 µM, 0.5 µM). A positive control with 5 µL DMSO and a negative control without enzyme were performed alongside every measurement. After incubation for 10 min at room temperature, 5 µL of 1 mM fluorogenic substrate in DMSO (Ac-Thz-Tle-Leu-Gln-AMC, *Peptides International*) were added and fluorescence (λ_{ex} = 360 nm,

λ_{em} = 460 nm) was measured at 37 °C every 30 s over 30 min in a Tecan M200 infinite Pro plate reader with 10 s of vigorous shaking before each measurement.

In order to determine activity at different enzyme concentrations, 90 µL of purified recombinant t3CL^{pro} or n3CL^{pro} (*Sigma-Aldrich*) in Tris-buffer (50 mM Tris-HCl, 1 mM EDTA, pH = 7.3) were mixed with 5 µL DMSO and 5 µL of 1 mM fluorogenic substrate in DMSO (Ala-Val-Leu-Gln-AMC, >98 % purity, *Thermo Fisher* or Ac-Thz-Tle-Leu-Gln-AMC, *Peptides International*) was added (final t3CL^{pro} and n3CL^{pro} concentrations: 500 nM, 250 nM, 100 nM, 50 nM, 25 nM). Fluorescence (λ_{ex} = 360 nm, λ_{em} = 460 nm) was measured at 37 °C every 30 s over 30 min in a Tecan M200 infinite Pro plate reader with 10 s of vigorous shaking before each measurement.

To test the enzymatic activity at different substrate concentrations, 5 µL of 2 mM purified recombinant t3CL^{pro} or pure n3CL^{pro} (*Sigma-Aldrich*) were diluted in 85 µL Tris-buffer (50 mM Tris-HCl, 1 mM EDTA, pH = 7.3). 5 µL DMSO and 5 µL of fluorogenic substrates in DMSO (Ala-Val-Leu-Gln-AMC, >98 % purity, *Thermo Fisher* or Ac-Thz-Tle-Leu-Gln-AMC, *Peptides International*) were added to the respective concentrations (250 µM, 125 µM, 50 µM, 25 µM, 12.5 µM, 5 µM). A negative control without enzyme and 125 µM substrate was run alongside each replicate. Fluorescence (λ_{ex} = 360 nm, λ_{em} = 460 nm) was measured at 37 °C every 30 s over 30 min in a Tecan M200 infinite Pro plate reader with 10 s of vigorous shaking before each measurement.

IC_{50} values: For each concentration of inhibitor, the enzymatic turnover rate [1/s] was determined by linear regression, whereby the first 5 min of fluorescence measurement were excluded for the data sets with PL^{pro}. Average percentages of enzyme activity and corresponding standard deviations were calculated and plotted against the logarithm of inhibitor concentration. Subsequent sigmoidal fit using GraphPad Prism gave the respective IC_{50} values.

6. Preparation of root extracts from red sage (*Salvia miltiorrhiza*)

For preparation of crude extracts, a protocol for purification of salvianolic acids was followed[4]. 10 g commercially available *Salvia miltiorrhiza* root powder was suspended in 200 mL 60 % ethanol (20 mL per 1 g powdered root) and subjected to sonication for 1 h. The mixture was filtered and the flow-through was concentrated *in vacuo* below 40 °C. Subsequent lyophilization yielded 4.916 g of crude extract as a brown solid. To further concentrate the extract, 4.5 g crude extract were resuspended in 50 mL H_2O and acidified with HCl to pH = 2. The mixture was extracted five times with ethyl acetate, the combined organic phases were dried over $MgSO_4$ and the solvent was evaporated, yielding 0.535 g of a dark red solid.

7. Molecular docking

Molecular docking was carried out with AutoDock 4.2.6 and AutoDock Tools 1.5.6[5]. As the Ligands are covalently bound we applied the 'flexible side chain' covalent docking as described by Bianco et. al.[6]

SARS-CoV-2 main protease (PDB ID: 6YB7) structure was retrieved from the RCSB PDB database (www.rcsb.org). All files required for docking of proteins and ligands were prepared by adapted "addcovalent" scipts provided by the authors of the covalent docking method utilizing AutoDock Tools 1.5.6 (ADT). The receptor was obtained from the PDB file by removing all waters and co-factors, adding hydrogens and calculating Gasteiger charges. All synthesized compounds were drawn in Chem Draw Professional 17.0 with the CS atoms of covalently bound cysteine and exported as smiles code. Maestro (Schrödinger Release 2019-2: Maestro, Schrödinger, LLC, New York, NY, 2020) was used to generate 3D structures from smiles and perform structure minimization/optimization with the OPLS 2005 force field[7]. In the flexible side chain method, two receptor atoms added to the ligand coordinates are used

to superimpose the ligand on the appropriate residue in the target protein. ADT was used to add hydrogens, calculate Gasteiger charges, and generate a modified flexible ligand, using default methods. During docking the resulting side chain–ligand structure is treated as flexible, allowing optimization of the interaction of the tethered ligand with the rest of the protein. Grid maps were calculated following the standard AutoDock protocol for flexible side chains. The Lamarckian Genetic Algorithm was used with default settings, generating 10 poses. 5 independent docking simulations have been performed for every ligand.

The structural models of the *N*-terminal $3CL^{pro}$-Strep-tag construct ($t3CL^{pro}$) and the corresponding native protein before autocleavage have been modeled with the comparative modeling software MODELER[8] by extending the SARS-CoV-2 main protease dimer structure (PDB ID: 6Y2E[9]) by the *N*-terminal part. 50 models have been constructed for every protein by applying the adjusted automodel scheme where only the new *N*-terminal part with 3 connected residues of the x-ray structure was optimized in order to stay as close as possible to the reference structure 6Y2E. The 10 best DOPE (Discrete Optimized Protein Energy)[10] scored models are then used to identify the covered part of the binding pocket of SARS-CoV-2 main protease.

Molecular graphics were produced with UCSF Chimera[11], developed by the Resource for Biocomputing, Visualization, and Informatics at the University of California, San Francisco, with support from NIH P41-GM103311. The visualization movie was generated with blender (http://www.blender.org) and VMD (visual molecular dynamics)[12].

References

1. Polic, V. & Auclair, K. Allosteric Activation of Cytochrome P450 3A4 via Progesterone Bioconjugation. *Bioconjug Chem* **28**, 885-889 (2017).
2. Beagle, L.K. et al. Efficient Synthesis of 2,5-Diketopiperazines by Staudinger-Mediated Cyclization. *Synlett*, 2337-2340 (2012).
3. Liu, Y. et al. Building Nanowires from Micelles: Hierarchical Self-Assembly of Alternating Amphiphilic Glycopolypeptide Brushes with Pendants of High-Mannose Glycodendron and Oligophenylalanine. *J Am Chem Soc* **138**, 12387-94 (2016).
4. Dong, J., Liu, Y., Liang, Z. & Wang, W. Investigation on ultrasound-assisted extraction of salvianolic acid B from Salvia miltiorrhiza root. *Ultrason Sonochem* **17**, 61-5 (2010).
5. Morris, G.M. et al. AutoDock4 and AutoDockTools4: Automated docking with selective receptor flexibility. *J Comput Chem* **30**, 2785-91 (2009).
6. Bianco, G., Forli, S., Goodsell, D.S. & Olson, A.J. Covalent docking using autodock: Two-point attractor and flexible side chain methods. *Protein Sci* **25**, 295-301 (2016).
7. Banks, J.L. et al. Integrated Modeling Program, Applied Chemical Theory (IMPACT). *J Comput Chem* **26**, 1752-80 (2005).
8. John, B. & Sali, A. Comparative protein structure modeling by iterative alignment, model building and model assessment. *Nucleic Acids Res* **31**, 3982-92 (2003).
9. Zhang, L. et al. Crystal structure of SARS-CoV-2 main protease provides a basis for design of improved alpha-ketoamide inhibitors. *Science* **368**, 409-412 (2020).
10. Shen, M.Y. & Sali, A. Statistical potential for assessment and prediction of protein structures. *Protein Sci* **15**, 2507-24 (2006).
11. Pettersen, E.F. et al. UCSF Chimera--a visualization system for exploratory research and analysis. *J Comput Chem* **25**, 1605-12 (2004).
12. Humphrey, W., Dalke, A. & Schulten, K. VMD: Visual molecular dynamics. *J Mol Graph* **14**, 33-38 (1996).

Tables

Supporting Table S1 Overview of the amine ligand library. (continued on following pages)

Ligand	Ligand Structure	Resulting ligand modified probe
**01**	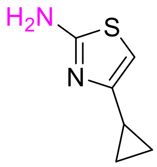	**LS01**
**02**	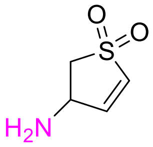	**LS02**
**03**	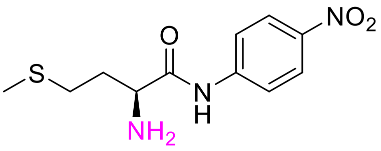	**LS03**
**04**	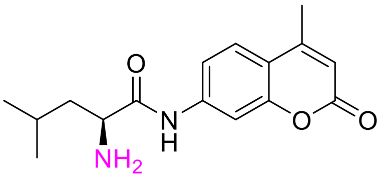	**LS04**
**05**	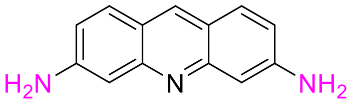	**LS05**
**06**	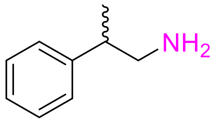	**LS06**
**07**	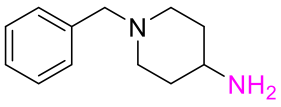	**LS07**
**08**	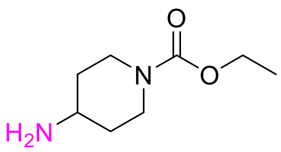	**LS08**
**09**	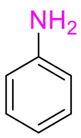	**LS09**

Supporting Table S1 (continued) Overview of the amine ligand library.

Ligand	Ligand Structure	Resulting ligand modified probe
**10**	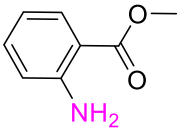	**LS10**
**11**	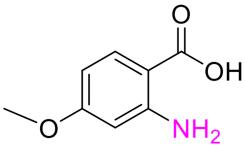	**LS11**
**12**	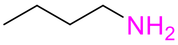	**LS12**
**13**	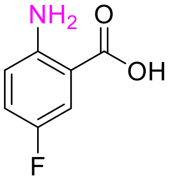	**LS13**
**14**	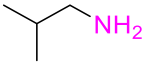	**LS14**
**15**	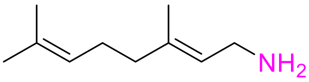	**LS15**
**16**	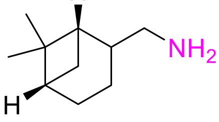	**LS16**
**17**	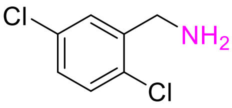	**LS17**
**18**	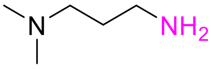	**LS18**

Supporting Table S1 (continued) Overview of the amine ligand library.

**Ligand**	**Ligand Structure**	**Resulting ligand modified probe**
**19**	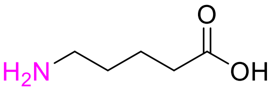	**LS19**
**20**	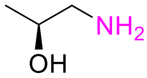	**LS20**
**21**	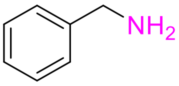	**LS21**
**22**	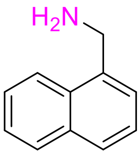	**LS22**
**23**	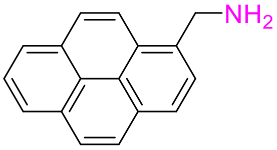	**LS23**
**24**	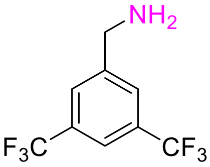	**LS24**
**25**	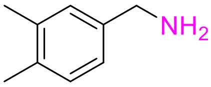	**LS25**
**26**	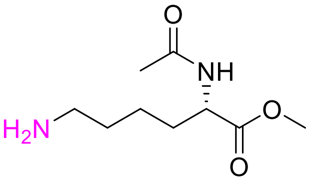	**LS26**
**27**	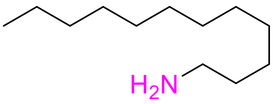	**LS27**

Supporting Table S2 Overview of electrophilic compounds used for competitive profiling.

**Aldehydes**		**CAS Number**	**Molecular Mass [g/mol]**
**A01**	Methyl 2-pyrrolyl ketone	1072-83-9	109.13
**A02**	Acetyl benzoyl	479-07-7	148.16
**A03**	Cyclamen aldehyde	103-95-7	190.28
**A04**	Ocean propanal	1205-17-0	192.21
**A05**	3,5,5-Trimethylhexanal	5435-64-3	142.24
**A07**	Vanillin	121-33-5	152.15
**A08**	Vanillin isobutyrate	20665-85-4	222.24
**Epoxides**			
**E01**	Ethyl 3-phenylglycidate	121-39-1	192.21
**Michael acceptors**			
**M01**	Cichoric acid	6537-80-0	474.37
**M02**	3,4-Dicaffeoylquinic acid	905-99-7	354.31
**M03**	Curcumin	458-37-7	368.38
**M04**	Filbertone	102322-83-8	126.2
**M05**	Benzylideneacetone	122-57-6	146.19
**M06**	Damascenone	23696-85-7	190.28
**M07**	Piperine	94-62-2	285.34
**M08**	Isopherone	78-59-1	138.21
**M09**	*trans*-3-(2-Furyl)acrolein	39511-08-5	122.12
**M10**	(1*R*)-(-)-Myrtenal	18486-69-6	150.22
**M11**	Cocoa hexenal	21834-92-4	188.27
**M12**	(*R*)-(+)-Pulegone	89-82-7	152.23
**M13**	(*S*)-(-)-Perillaldehyde	18031-40-8	150.22
**M18**	α-Hexylcinnamaldehyde	101-86-0	216.32
**M19**	α-Isomethyl Ionone	127-51-5	206.32
**M21**	3-Methyl-2-butenal	107-86-8	84.12
**M22**	5-Methyl-2-phenyl-2-hexenal	21834-92-4	188.27
**M23**	Methyl 2-octynoate	111-12-6	154.21
**M24**	4-Acetoxy-2,5-dimethyl-3(2H)furanone	4166-20-5	170.16
**M25**	Dihydrojasmone	1128-08-1	166.26
**M26**	Salvianolic acid B	121521-90-2	718.61
**M27**	Licochalcone A	58749-22-7	338.4
**M29**	Methyl *trans*-cinnamate	1754-62-7	162.19
**M30**	Ethyl cinnamate	103-36-6	176.21
**M31**	Isoamyl cinnamate	7779-65-9	218.29
**M32**	Isopropyl cinnamate	7780-06-5	190.24
**M33**	Salvianolic acid A	96574-01-5	494.45
**M34**	Salvianolic acid C	115841-09-3	492.4
**M35**	Rosmarinic acid	20283-92-5	360.31
**M36**	Lithospermic acid	28831-65-4	538.5
**M38**	L-Mimosine	500-44-7	198.18
**Protease Inhibitors**			
**P01**	Bestatin Hydrochloride	65391-42-6	344.83
**P02**	Nelfinavir	159989-64-7	567.78
**P03**	E-64	66701-25-5	357.41
**Others**			
**X01**	Cinnamyl isobutyrate	103-59-3	204.26
**X02**	Isoeugenyl acetate	93-29-8	206.24
**X03**	Maltyl isobutyrate	65416-14-0	196.2
**X04**	4-Methyl-1-phenyl-2-pentanone	5349-62-2	176.25
**X05**	2-Phenylethyl isothiocyanate	2257-09-2	163.24
**X06**	2-Acetyl-2-thiazoline	29926-41-8	129.18
**X07**	Furfuryl thioacetate	13678-68-7	156.2

Figures

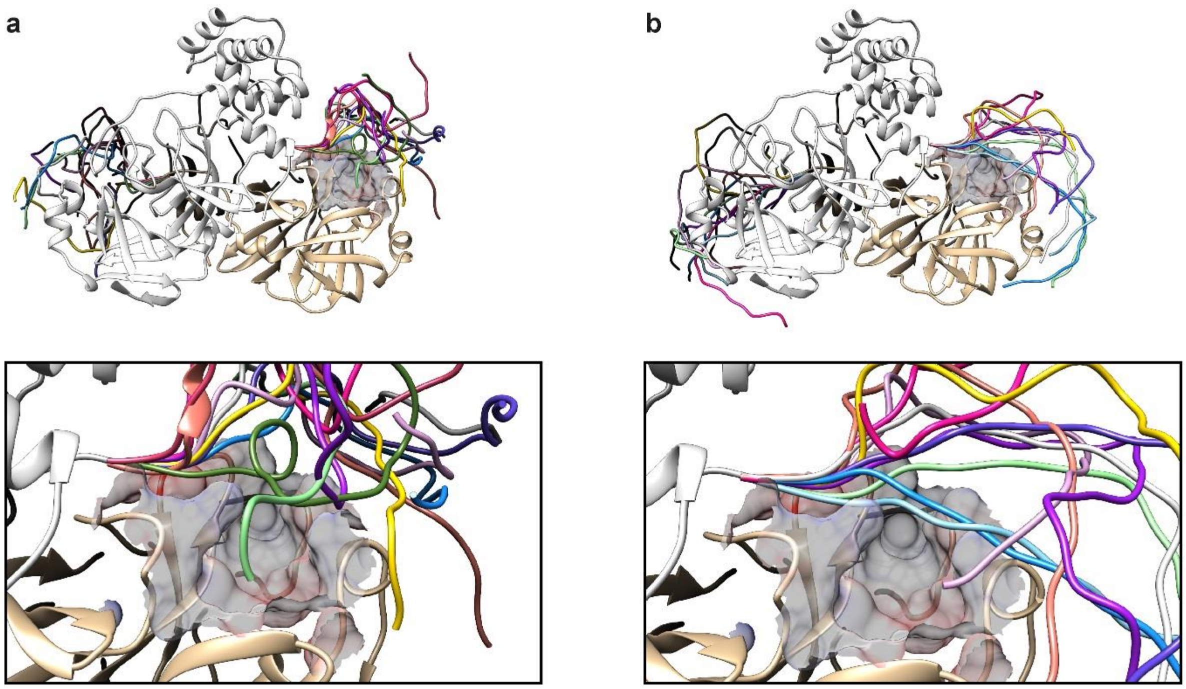

Fig. S1 Molecular modelling of the extended native *N*-terminus of 3CL^{pro} (PDB ID: 6Y2E[9]) with the best DOPE scored models of the *N*-terminal sequence overlaid in color. **a**, Model for the *N*-terminal Strep-tag II.

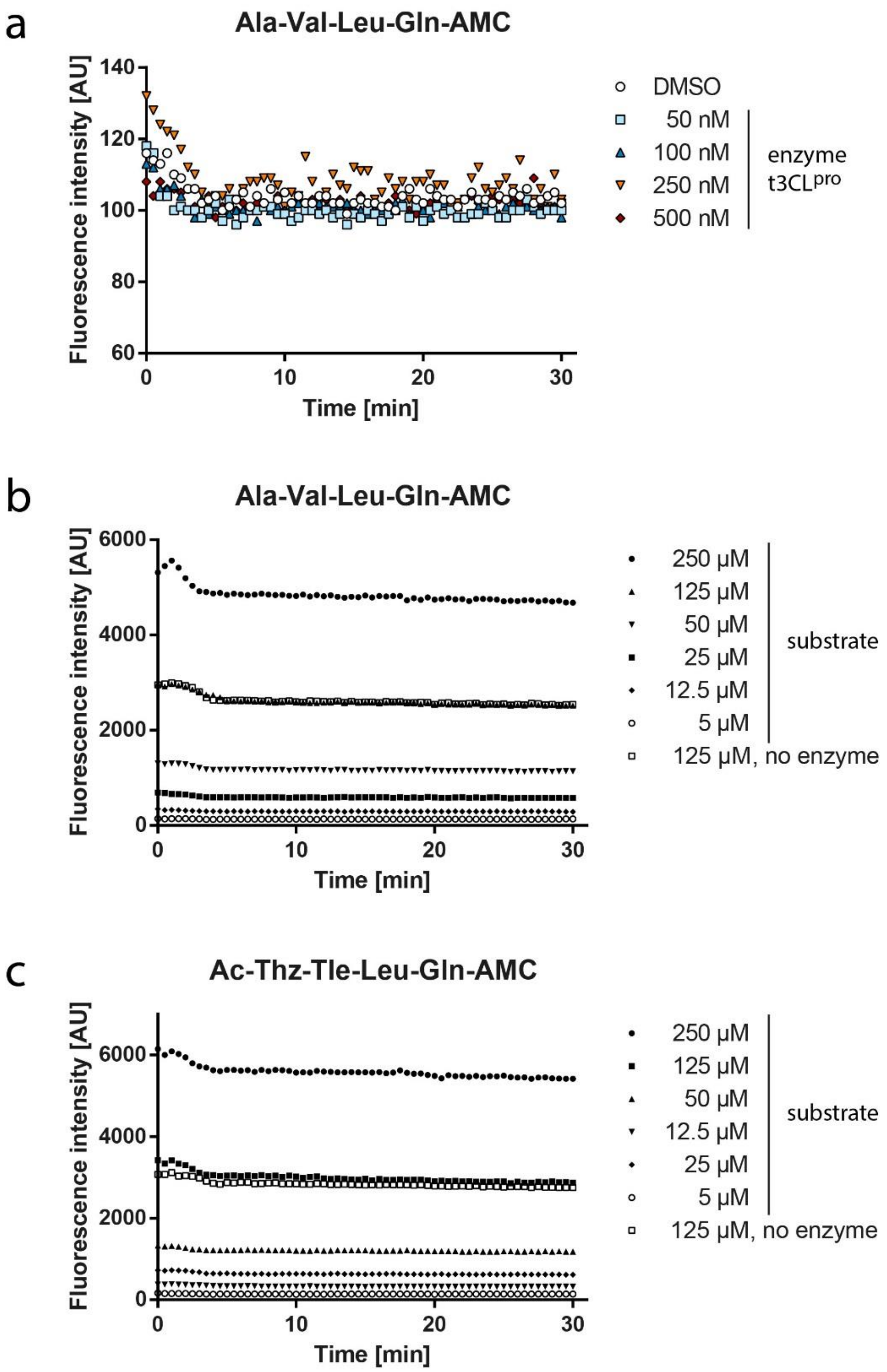

Fig. S2 Cleavage assays for purified t3CL^{pro} using fluorogenic (7-amino-4-methylcoumarin, AMC) peptide substrates. **a**, Using different enzyme concentrations from 50 nM to 500 nM. **b**, Using peptidic substrate concentrations between 5 µM and 250 µM. **c**, With a different peptidic substrate in the range from 5 µM to 250 µM. For **b** and **c** fluorescence intensities with and without enzyme overlap at 125 µM.

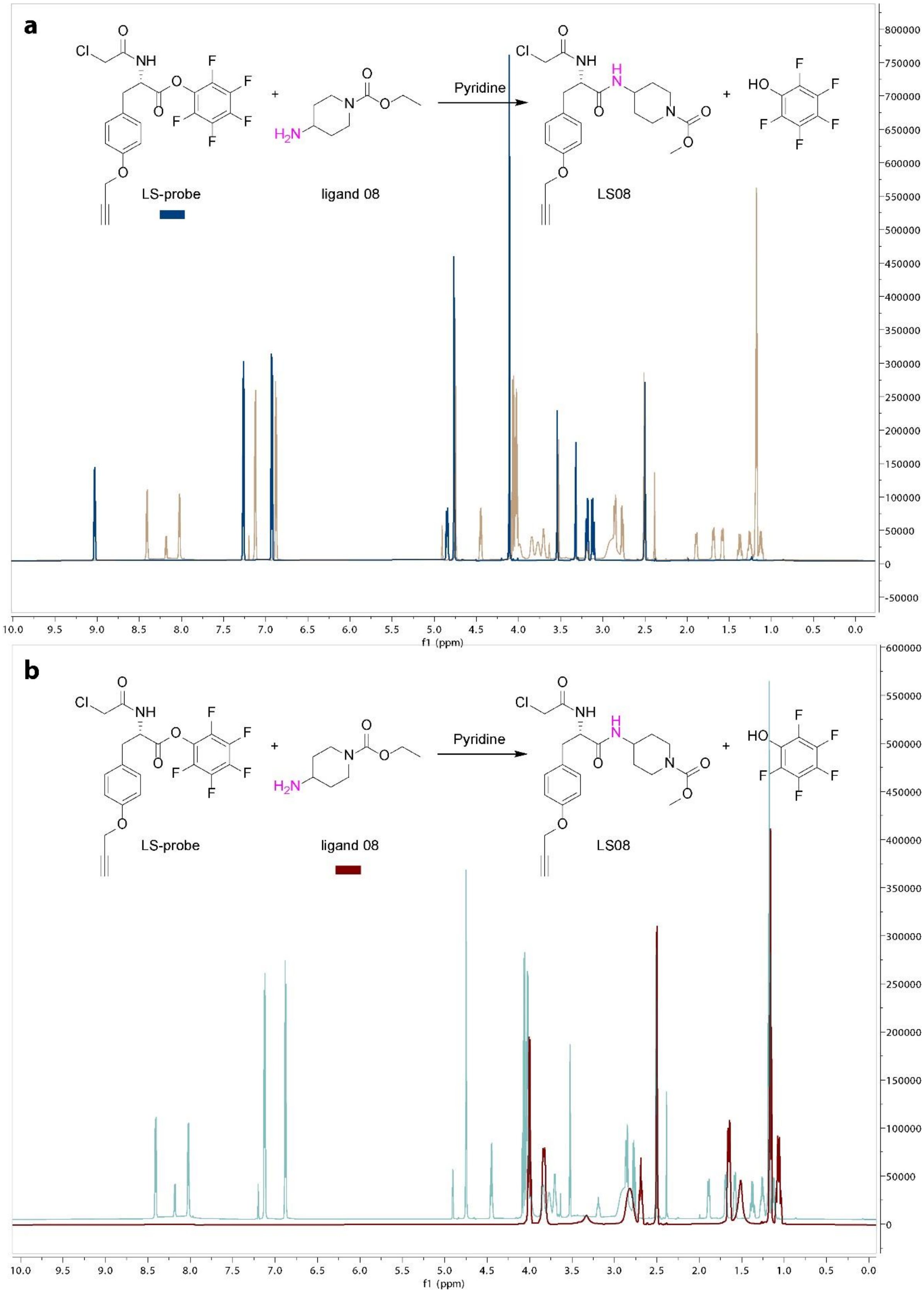

Fig. S3 Diversification reaction of the LS-probe with the ligand 08 (primary aliphatic amine) with pyridine is completed after 15 min as indicated by ^{1}H NMR spectroscopy in DMSO-d_6. **a**, overlaid spectra of the reaction (gold) and the unreacted LS-probe (blue). **b**, overlaid spectra of the reaction (turquoise) and the unreacted ligand (brown).

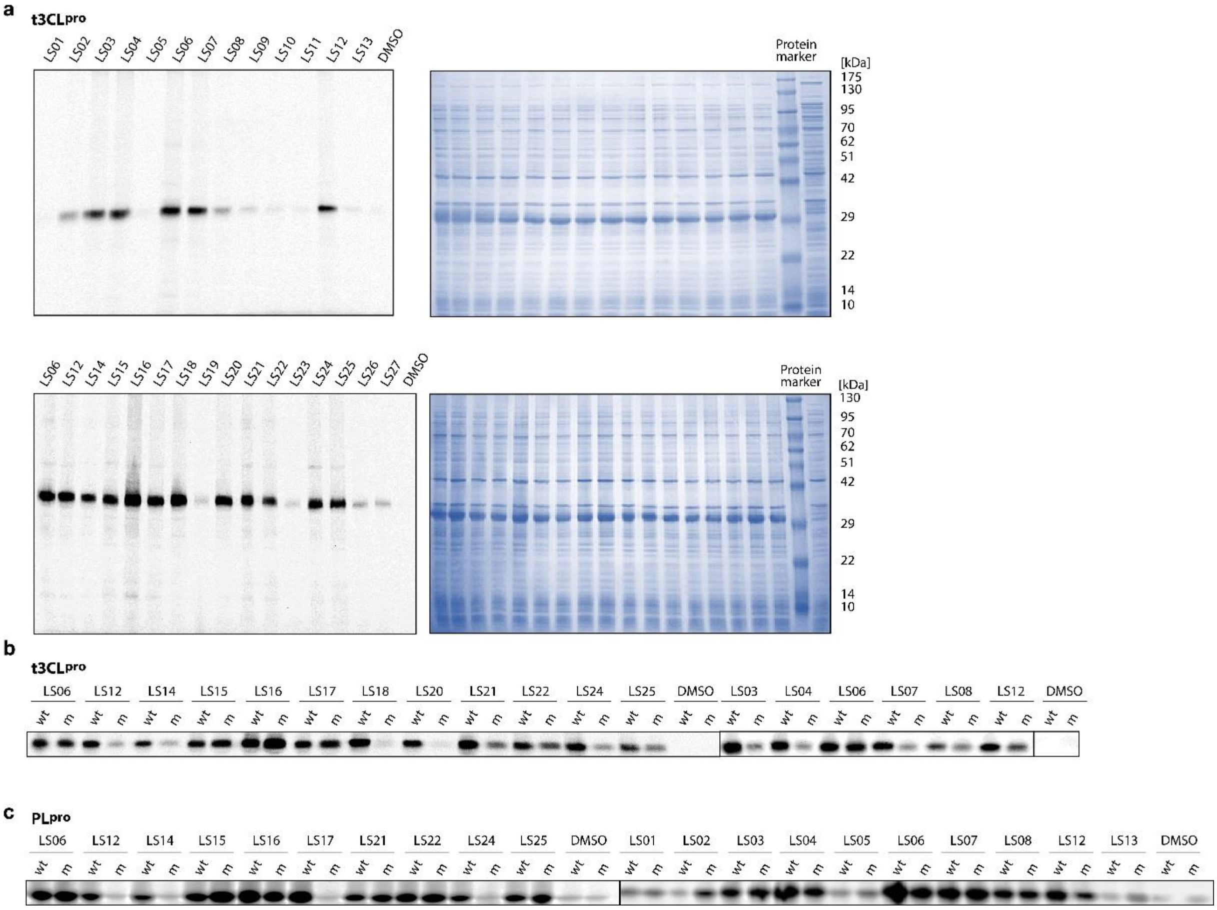

Fig. S4 Examples of gels for the *in situ* labelling of t3CL^{pro} and PL^{pro} by the LS probes. **a**, initial screening for active probes against t3CL^{pro}. **b**, Comparison of wild type (wt) and mutants (m) for t3CL^{pro}. **c**, Comparison of wild type (wt) and mutants (m) for PL^{pro}.

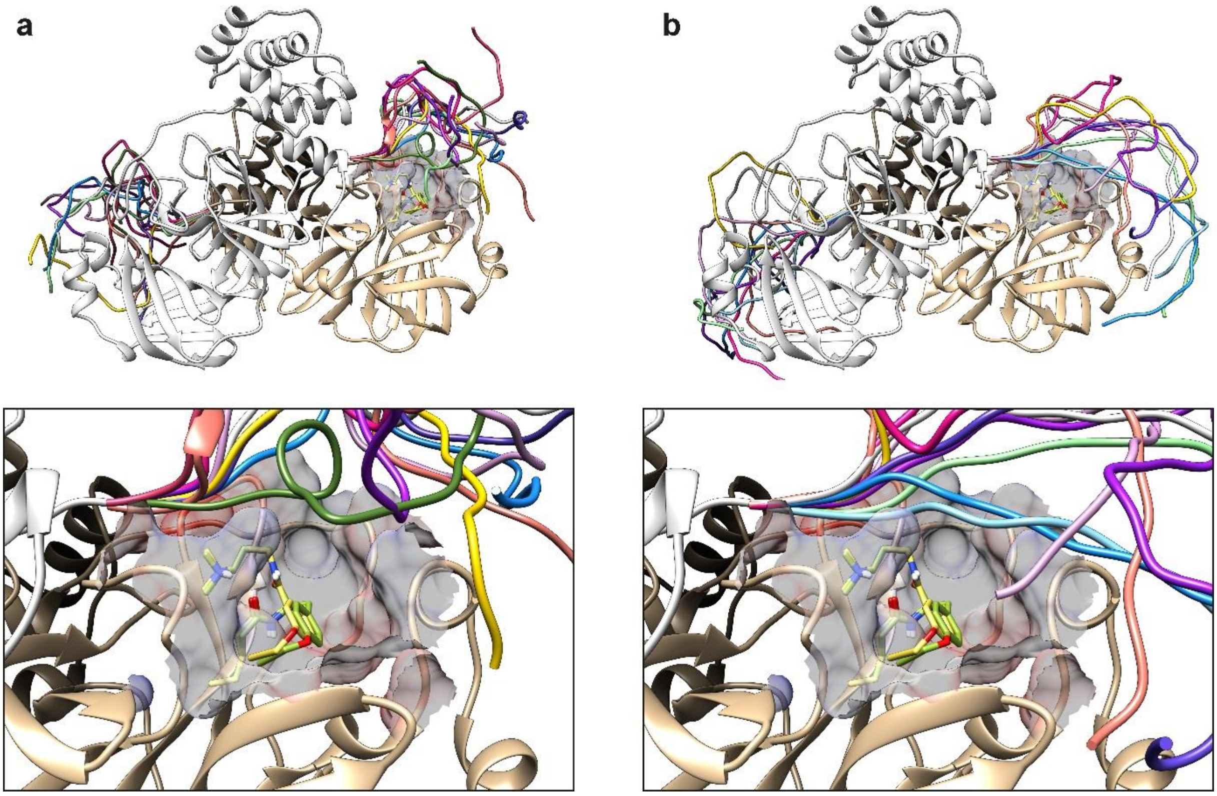

Fig. S5 Molecular docking of **LS18** into the model of 3CL^{pro} (based on PDB ID: 6Y2E[9]) featuring an extended N-terminus of Strep-tag II (t3CL^{pro}) (**a**) and wild type in the pre-activation stage before self-cleavage (**b**) sequence.

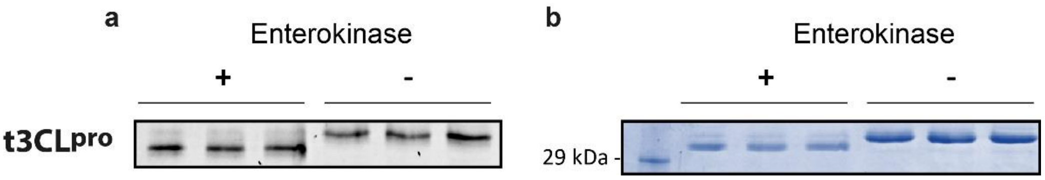

Fig. S6 t3CL^{pro} with the *N*-terminal strep tag cleaved by enterokinase (+) labelled by probe **LS18** in comparison to full length t3CL^{pro}. **a**, Fluorescence gel; **b**, Coomassie strained gel.

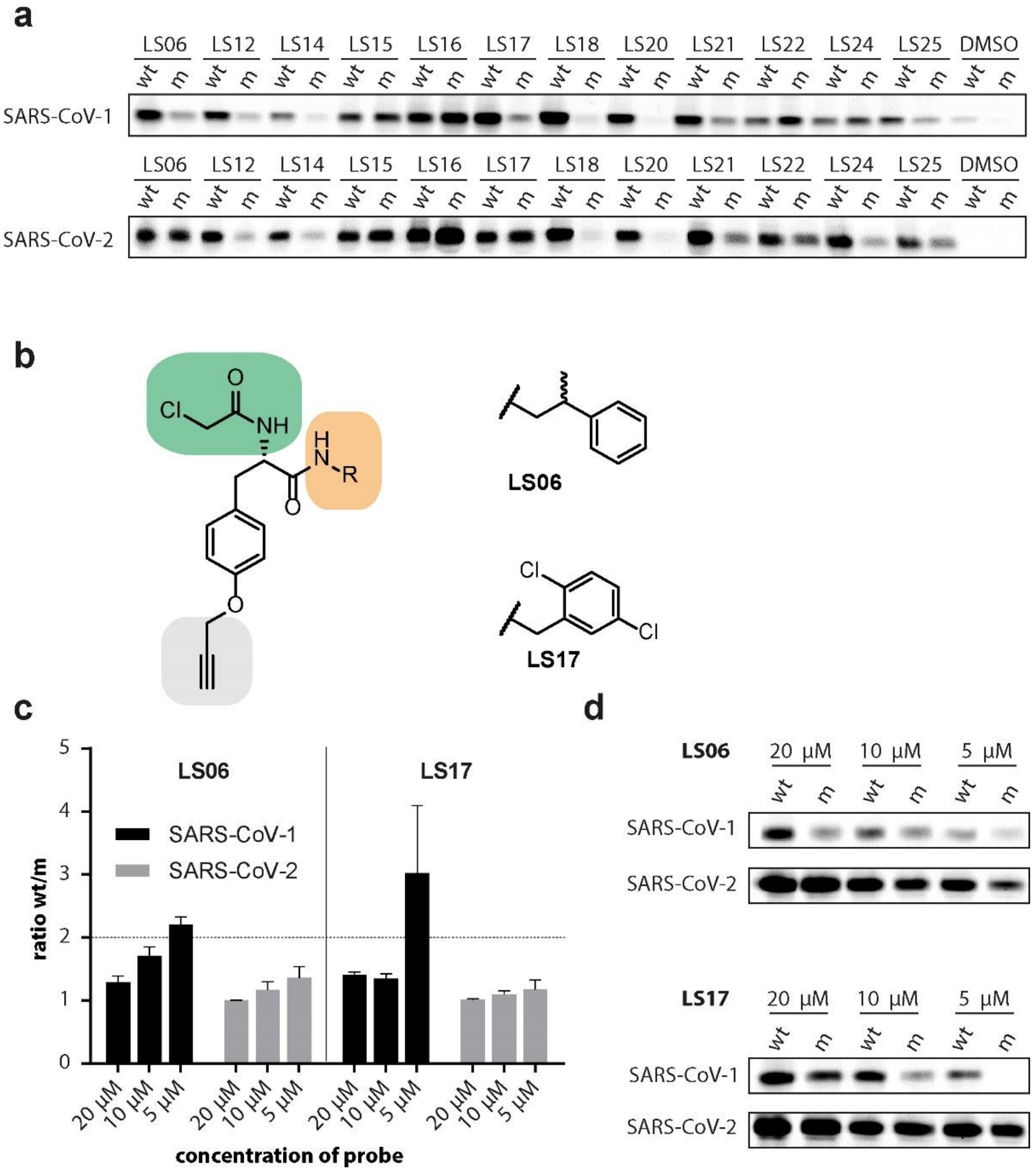

Fig. S7 Comparison of the *in situ* labelling of t3CL^{pro} of SARS-CoV-1 and SARS-CoV-2 in live heterologously expressing *E. coli* cells. **a**, Most probes yield equal labelling intensity and specificity for the two t3CL^{pro} homologues. **b**, Chemical structures of probes **LS06** and **LS17**. **c**, Specificity of probes **LS06** and **LS17** for labelling t3CL^{pro} of SARS-CoV-1 and SARS-CoV-2 given by the ratio of labelling intensities of wild type (wt) to mutant (m) (n=3). **d**, Exemplary fluorescence gels showing the labelling of t3CL^{pro} of SARS-CoV-1 and SARS-CoV-2 by different concentrations of the probes.

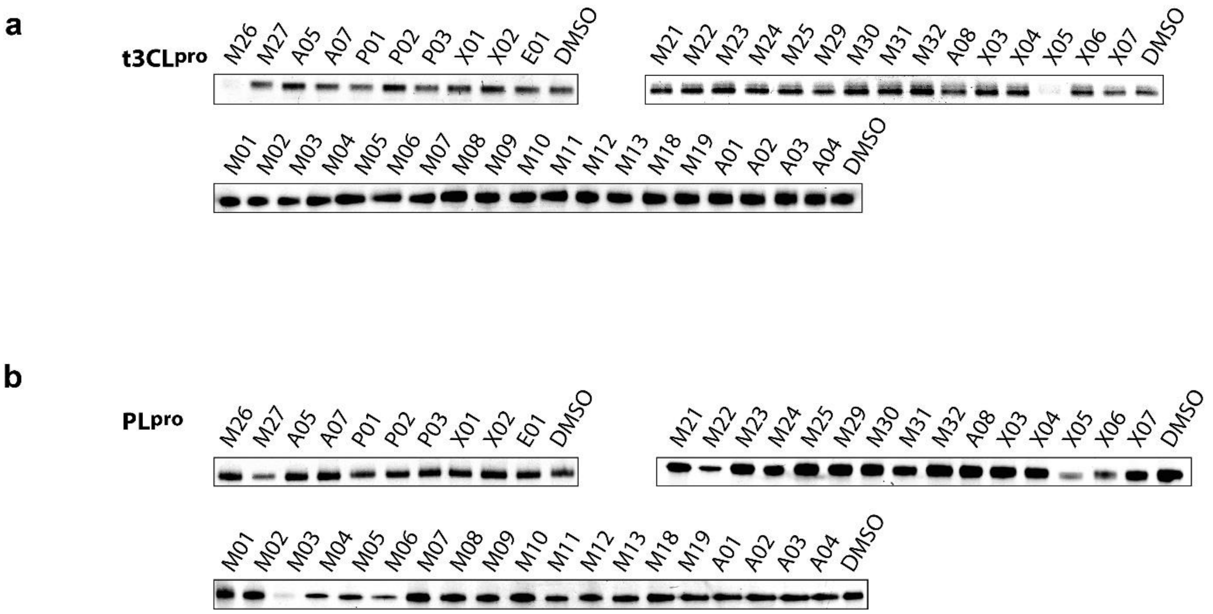

Fig. S8 Competitive profiling of the electrophilic compound library (Suppl. Table 2) at 200 µM against *in vitro* labelling of t3CL^{pro} (**a**) and (**b**) PL^{pro} by 5 µM probe **LS18** and **LS24**, respectively. Representative gels of three independent replicates.

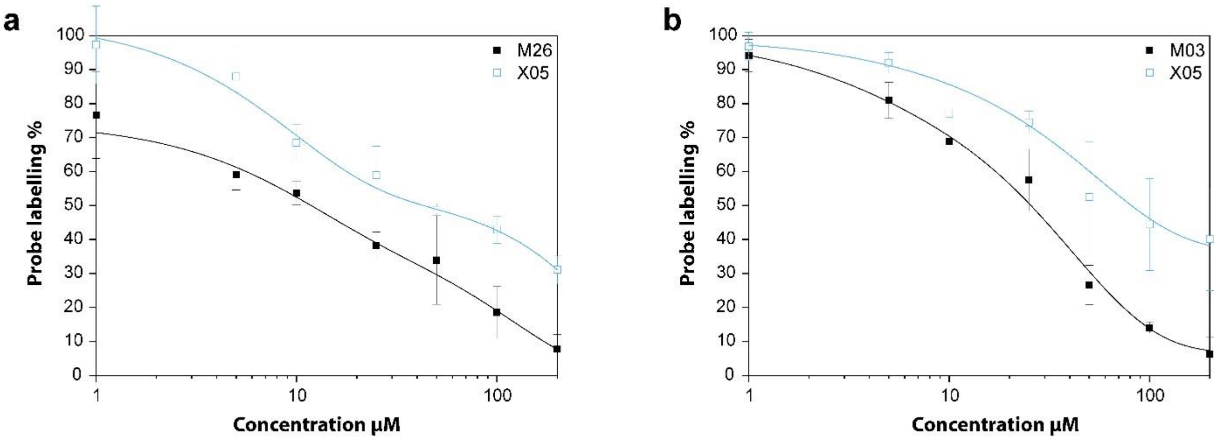

Fig. S9 Half-maximal inhibitory concentrations (IC_{50}s) determined from curve fittings of quantitative competitive labelling experiments. **a**, With compounds **M26** and **X05** inhibiting labelling of t3CL^{pro} by probe **LS18** (IC_{50} = 12 μM for **M26** = **SalB**; IC_{50} = 43 μM for **X05**). **b**, With compounds **M03** and **X05** inhibiting labelling of PL^{pro} by probe **LS24** (IC_{50} = 26 μM for **M03**; IC_{50} = 81 μM for **X05**).

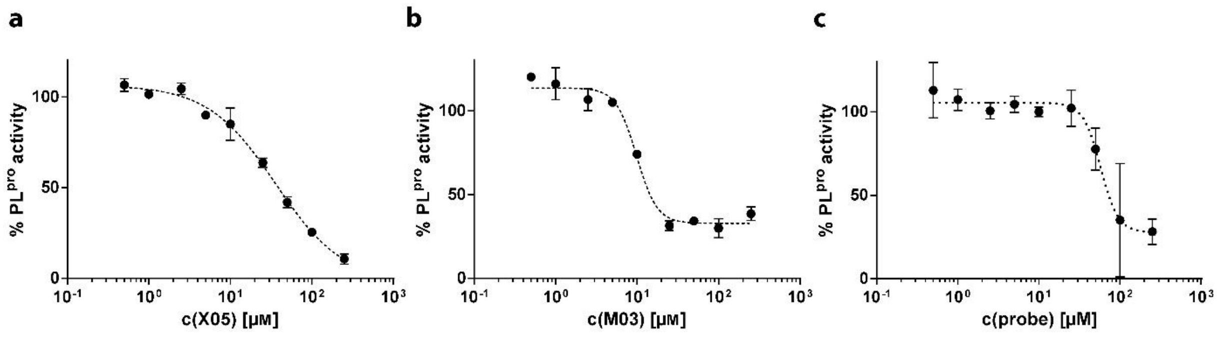

Fig. S10 Inhibition of PL^{pro} determined by an enzyme activity assay using the fluorogenic substrate Arg-Leu-Arg-Gly-Gly-AMC. **a**, Inhibition of PL^{pro} activity by **X05** (IC_{50} = 44 μM). **b**, Inhibition of PL^{pro} activity by **M03** (IC_{50} = 10 μM). **c**, Inhibition of PL^{pro} activity by probe **LS24** (IC_{50} = 58 μM).

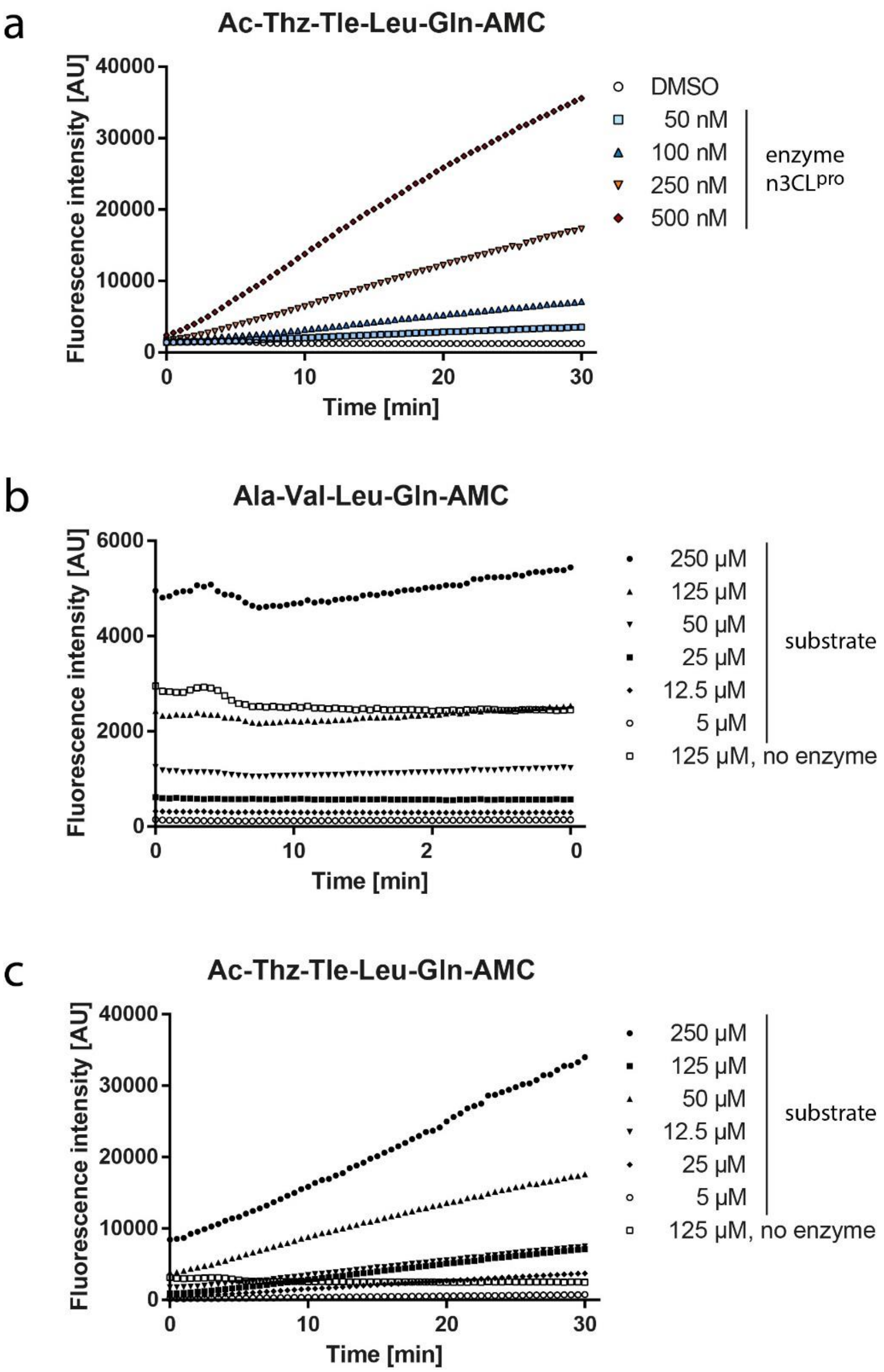

Fig. S11 Cleavage assays with n3CL^{pro} using fluorogenic (7-amino-4-methylcoumarin, AMC) peptide substrates. **a**, Using different enzyme concentrations from 50 nM to 500 nM. **b**, Using peptidic substrate concentrations between 5 µM and 250 µM. **c**, With a different peptidic substrate in the range from 5 µM to 250 µM. For **b** and **c** fluorescence intensities with and without enzyme overlap at 125 µM.

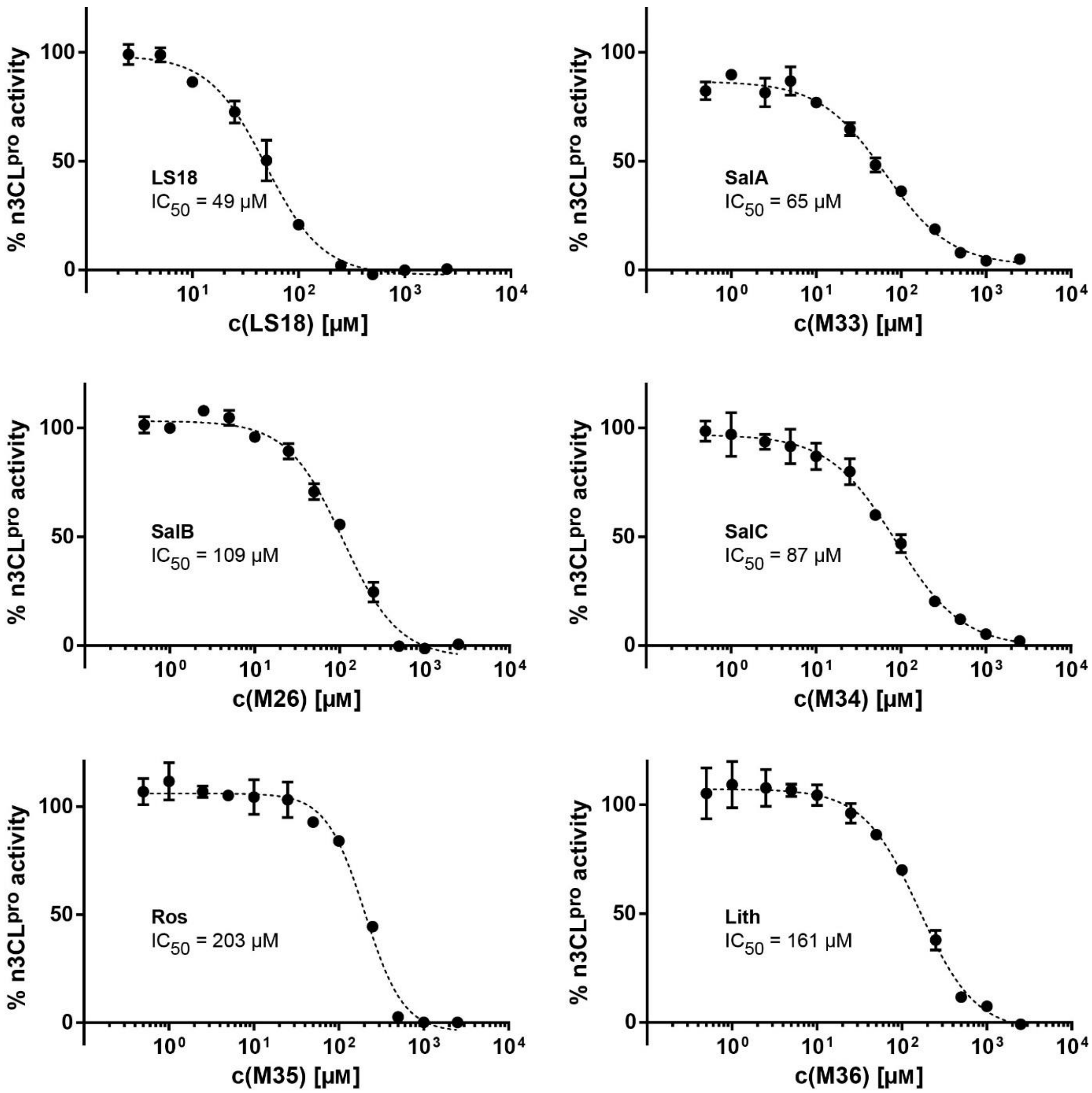

Fig. S12 Inhibition of n3CL^{pro} by **LS18** and the inhibitors **SalA**, **SalB**, **SalC**, **Ros**, and **Lith** determined by an enzyme activity assay using the fluorogenic substrate Ac-Thz-Tle-Leu-Gln-AMC.

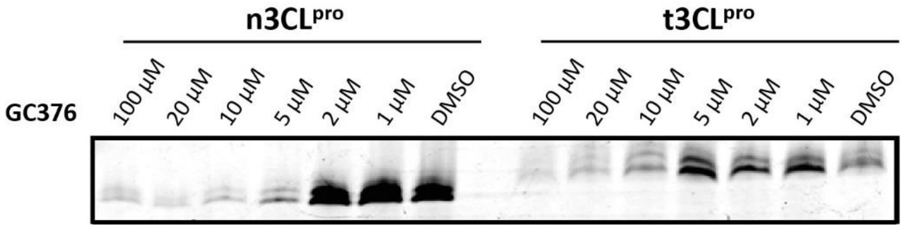

Fig. S13 Competitive profiling of the 3CL^{pro} inhibitor GC376 in the concentration range of 1-100 µM against n3CL^{pro} and t3CL^{pro} using probe **LS18**.

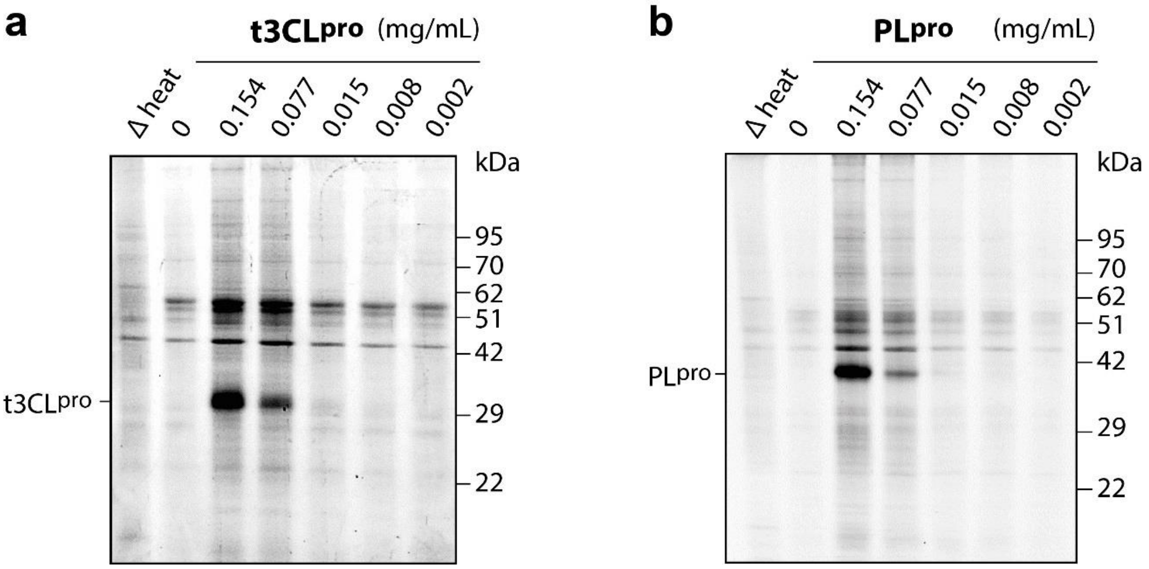

Fig. S14 Labelling of $t3CL^{pro}$ (**LS18**) and PL^{pro} (**LS24**) in the background of the native proteome of HepG2 cell lysates at 20 µM probe concentration. Controls (Δ heat) give the unspecific background labelling of the heat denatured proteome.

Annex: NMR Spectra

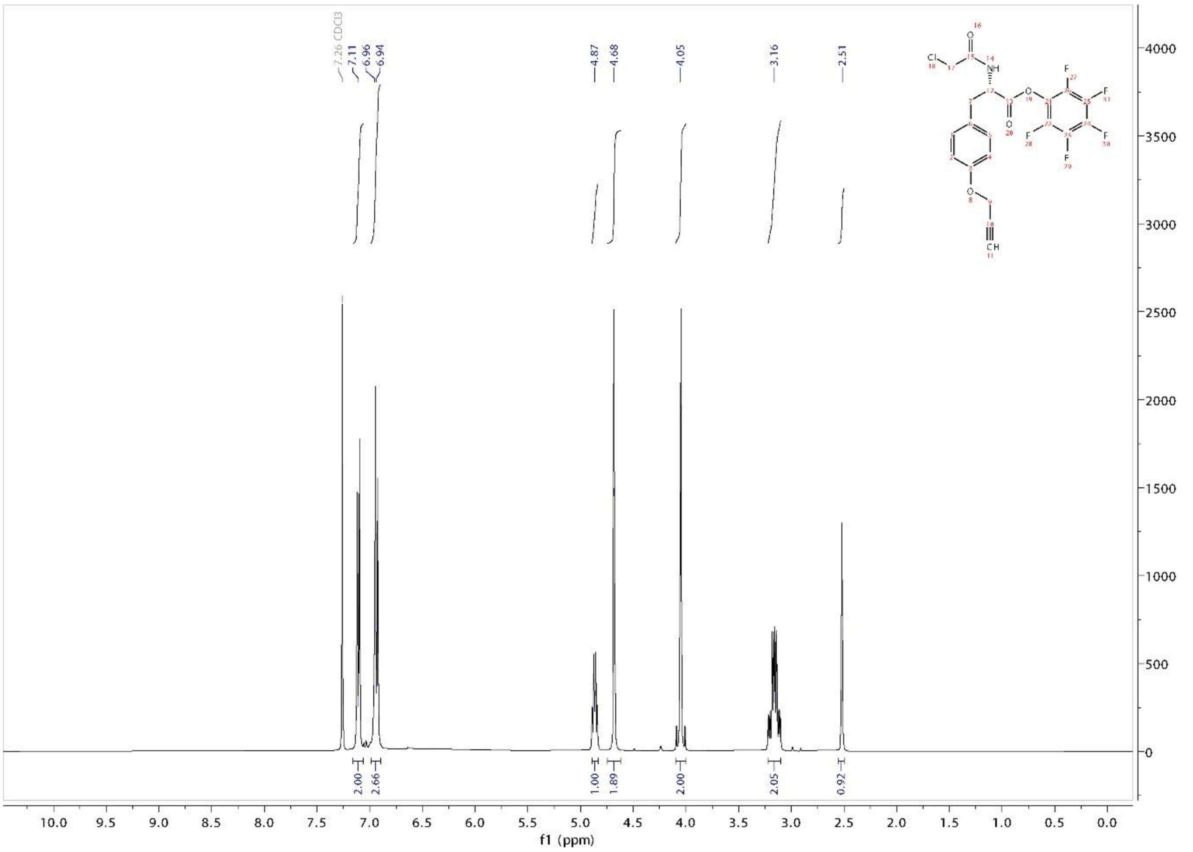

^{1}H-NMR spectrum of the LS-probe **(4)** in $CDCl_3$.

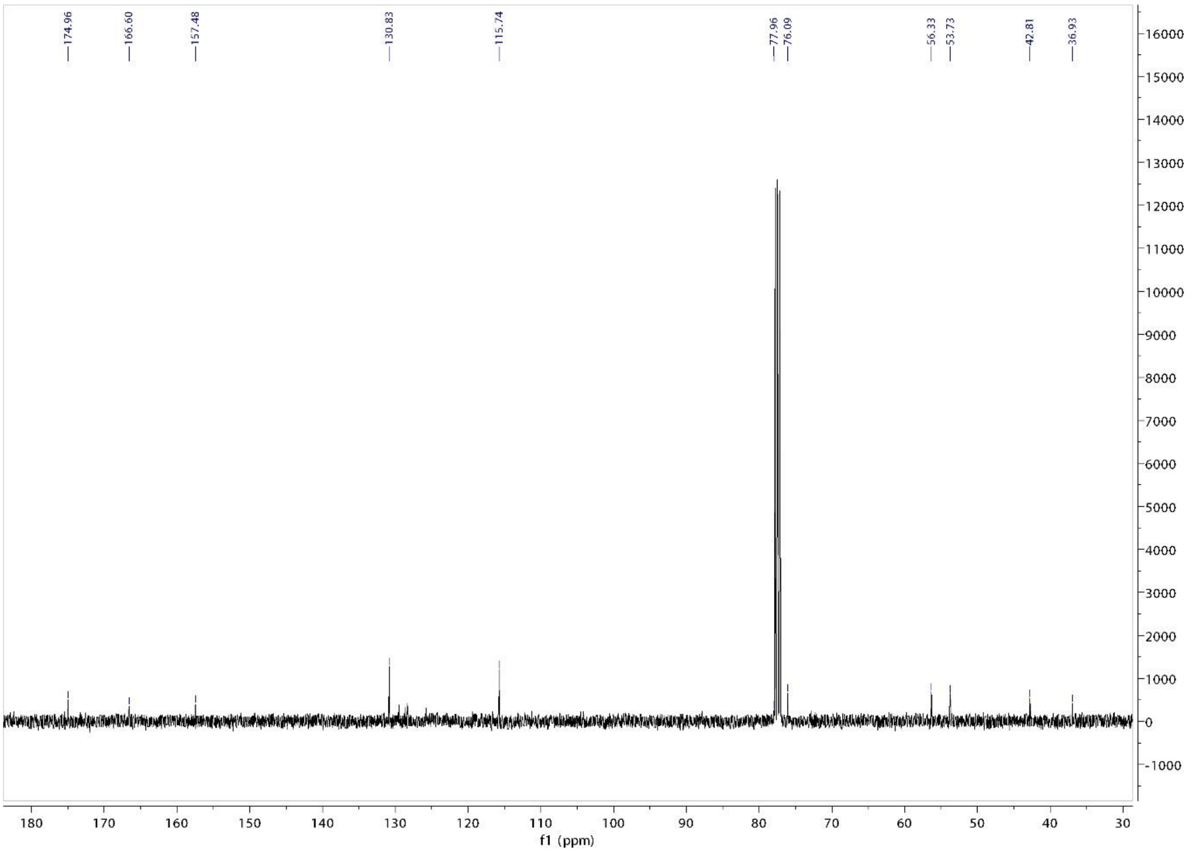

^{13}C-NMR spectrum of the LS-probe **(4)** in $CDCl_3$.

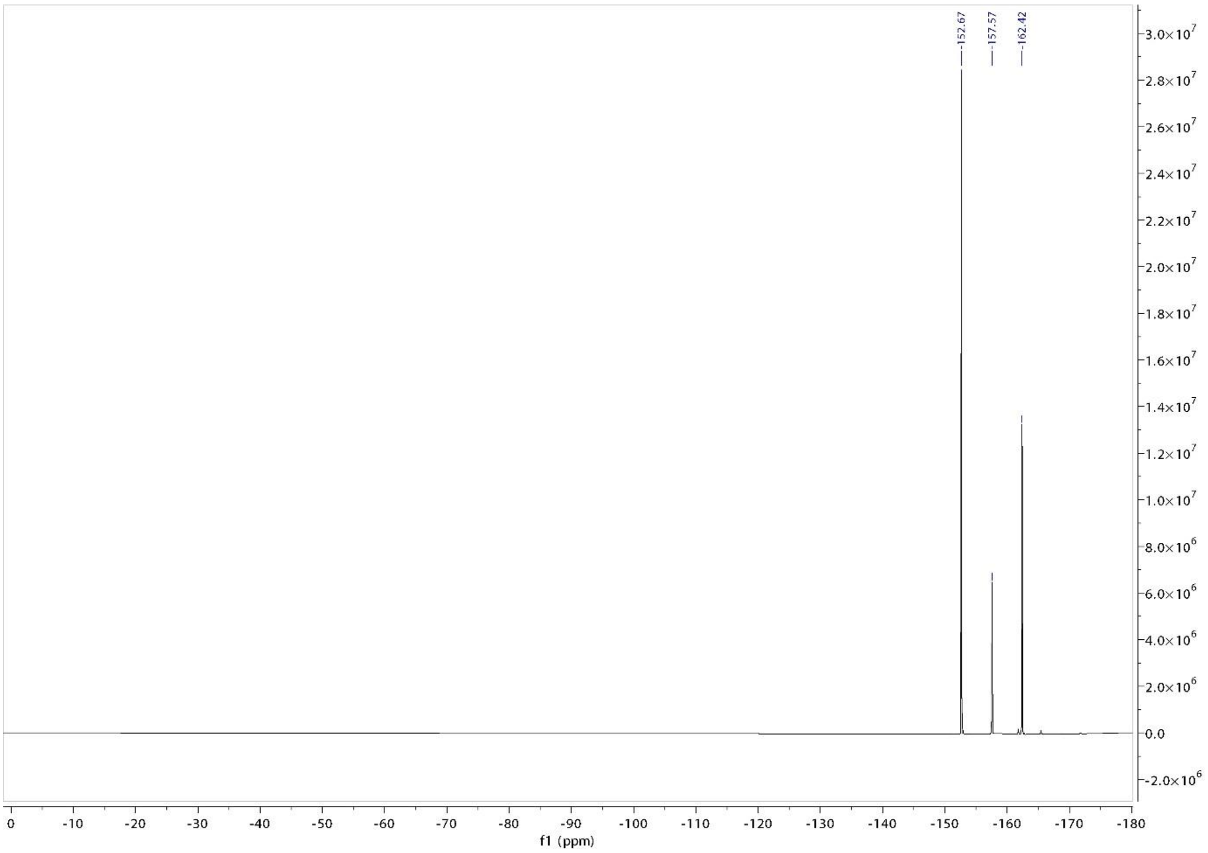

^{19}F-NMR spectrum of the LS-probe **(4)** in $CDCl_3$.

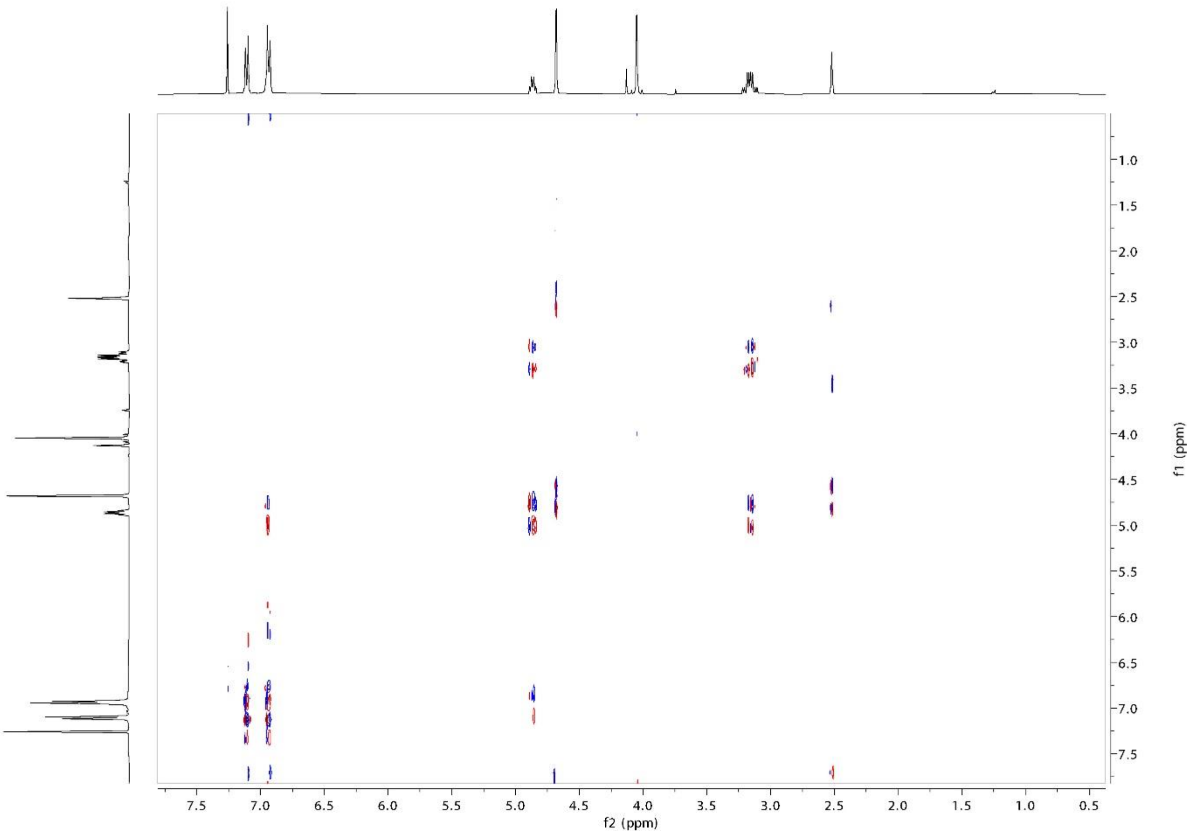

COSY-NMR spectrum of the LS-probe **(4)** in $CDCl_3$.

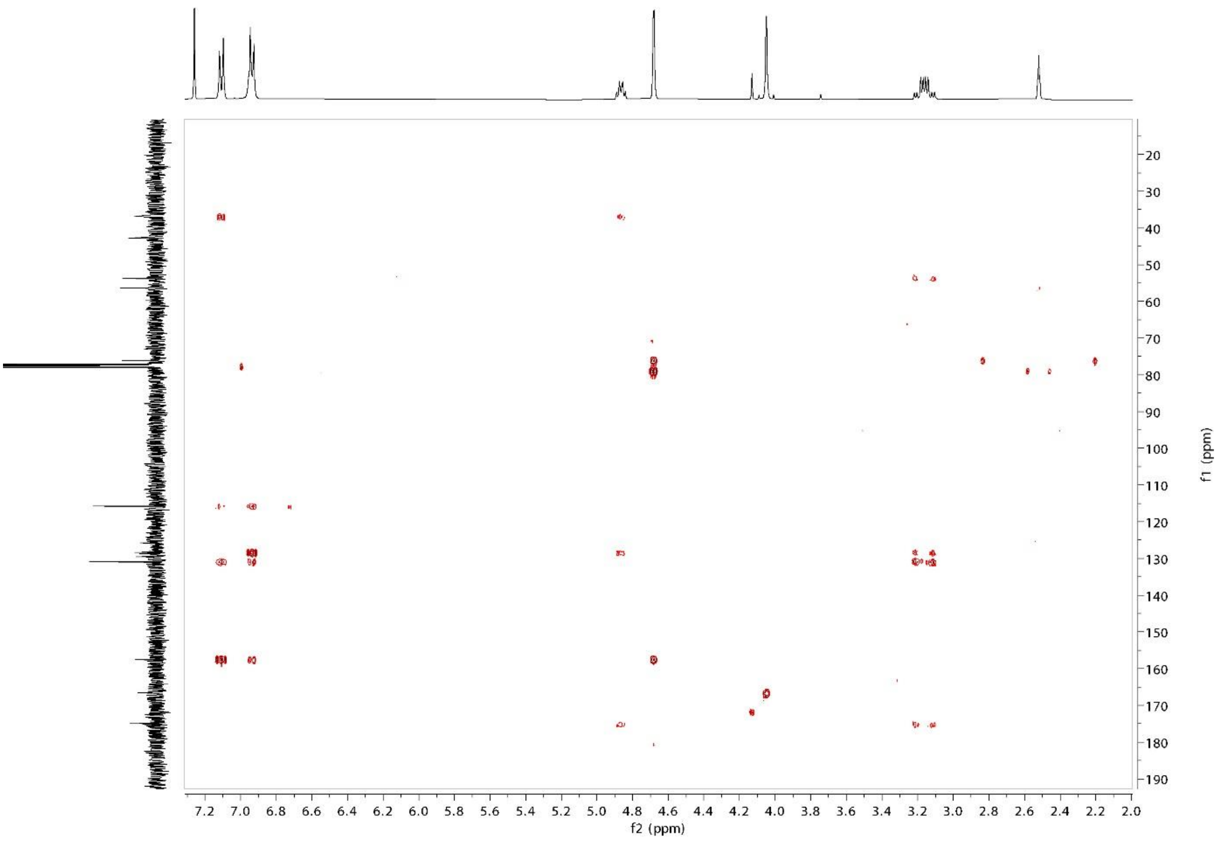

HMBC-NMR spectrum of the LS-probe **(4)** in $CDCl_3$.

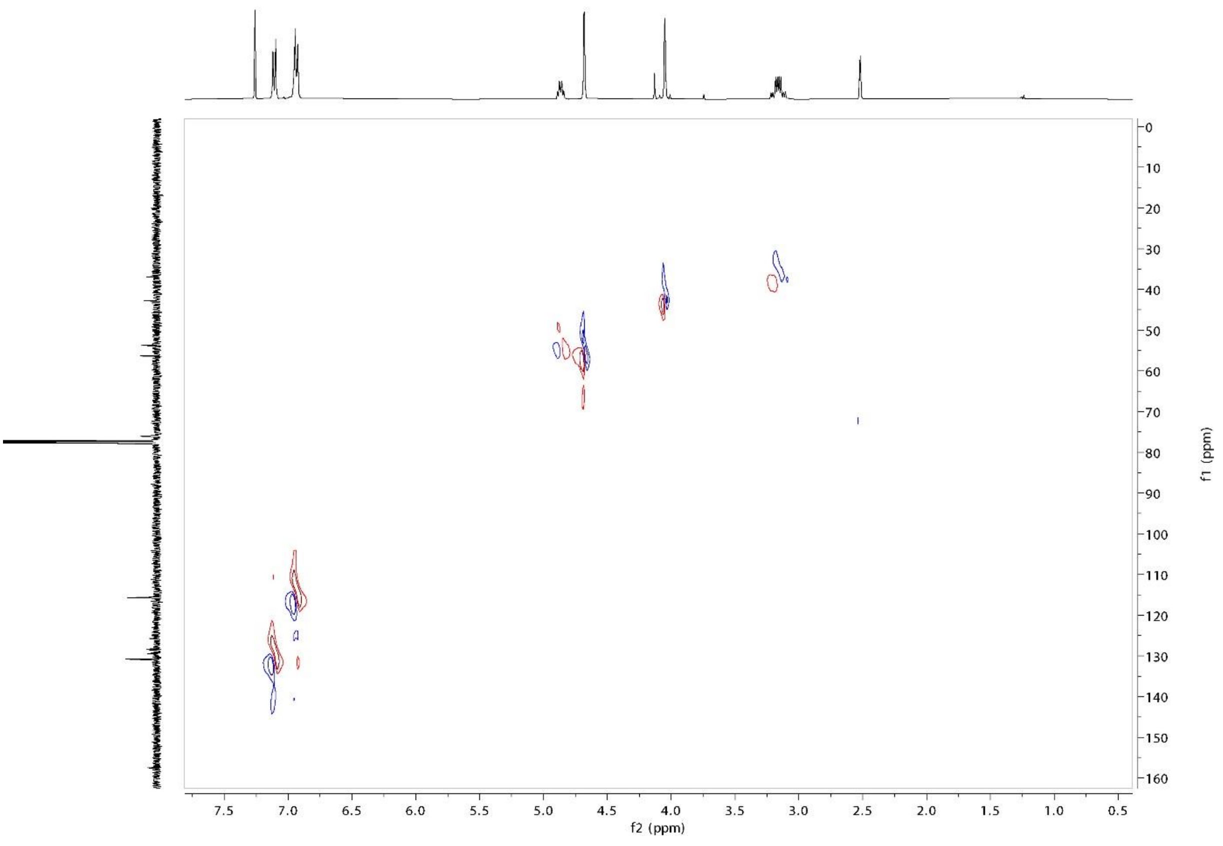

HSQC-NMR spectrum of the LS-probe **(4)** in $CDCl_3$.

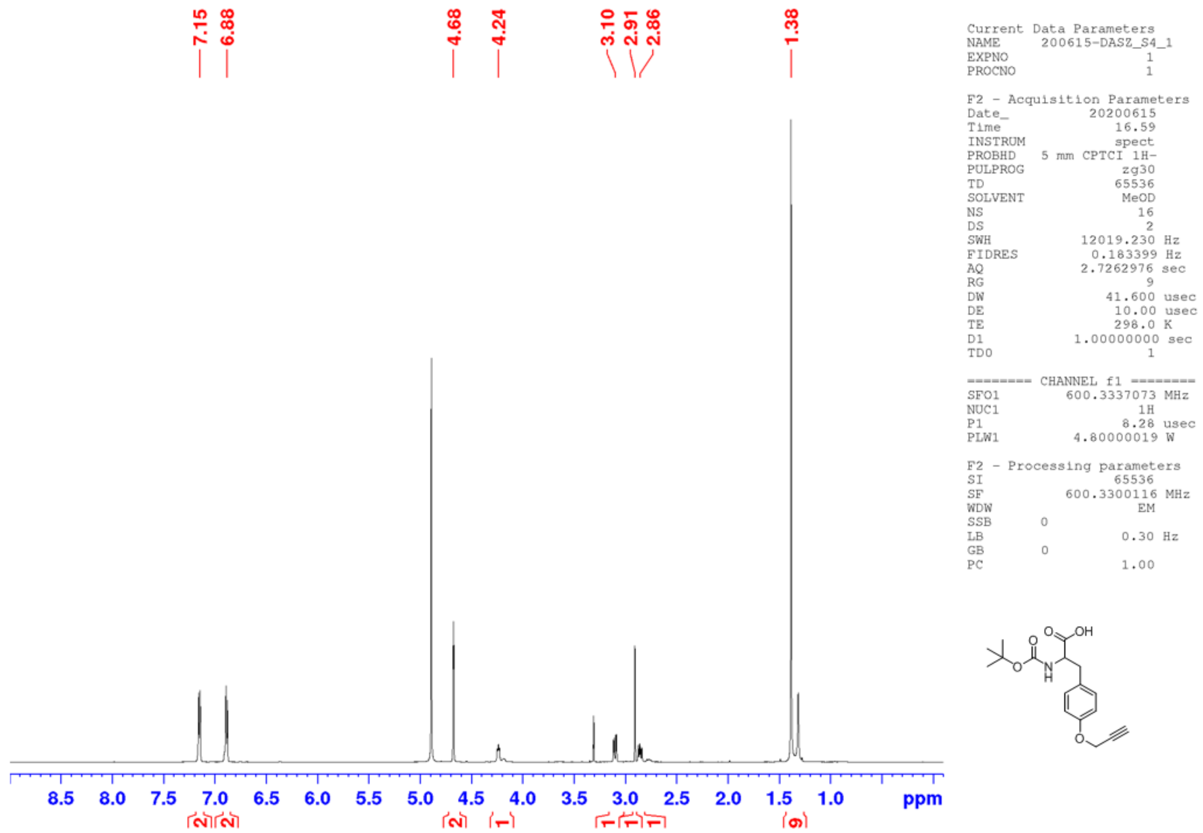

^{1}H-NMR spectrum of **(5)** in MeOD

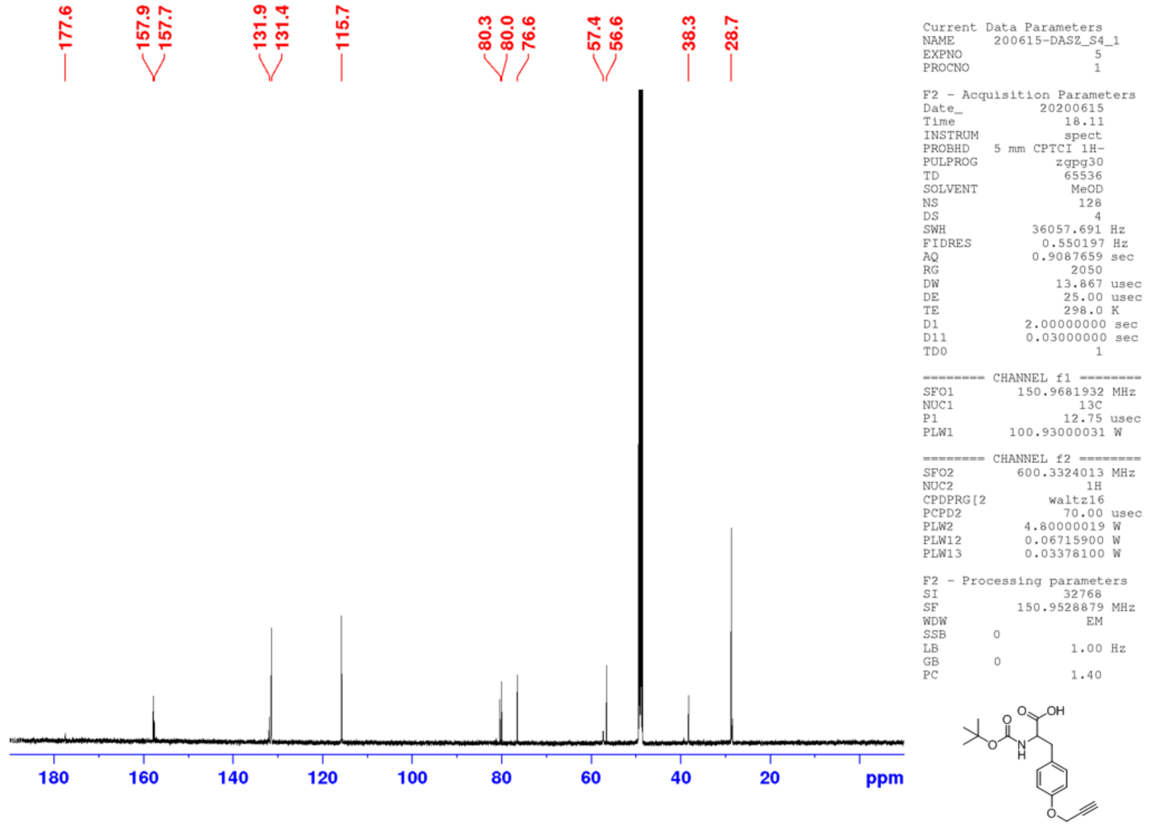

^{13}C-NMR spectrum of **(5)** in MeOD

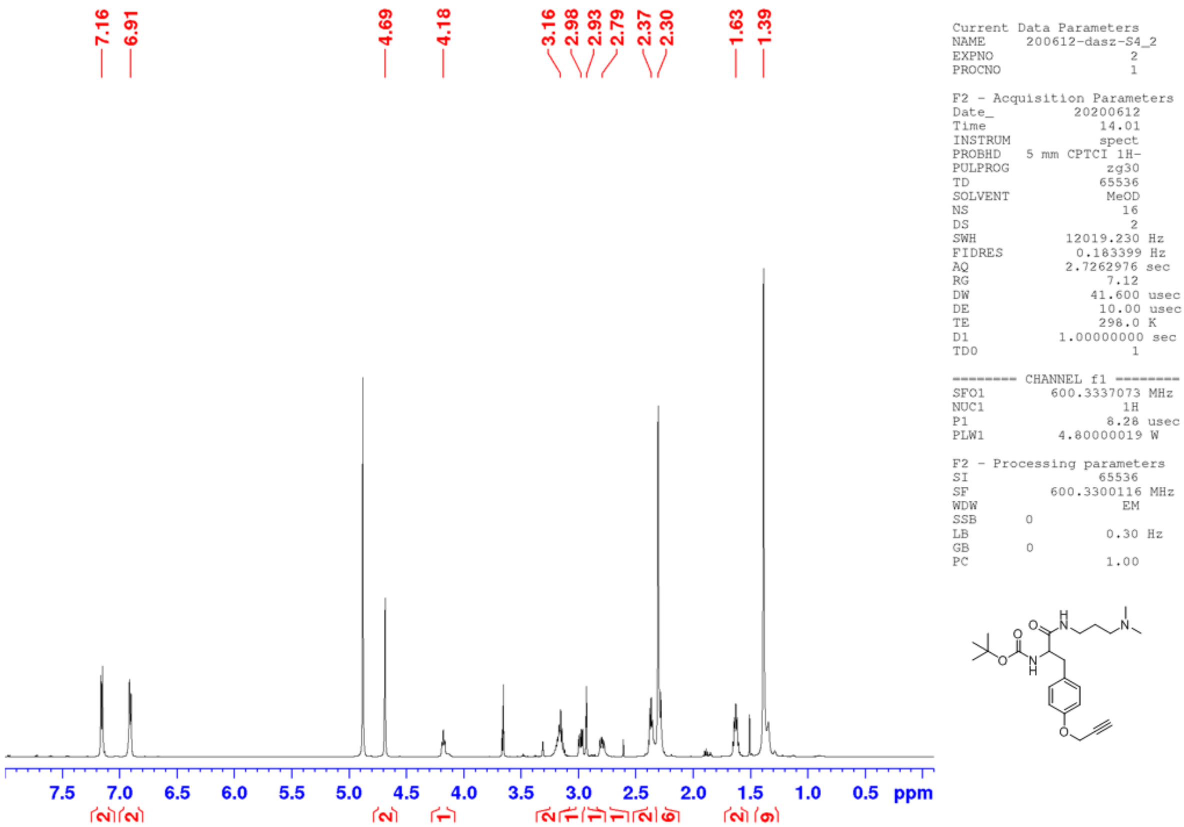

^{1}H-NMR spectrum of **(6)** in MeOD

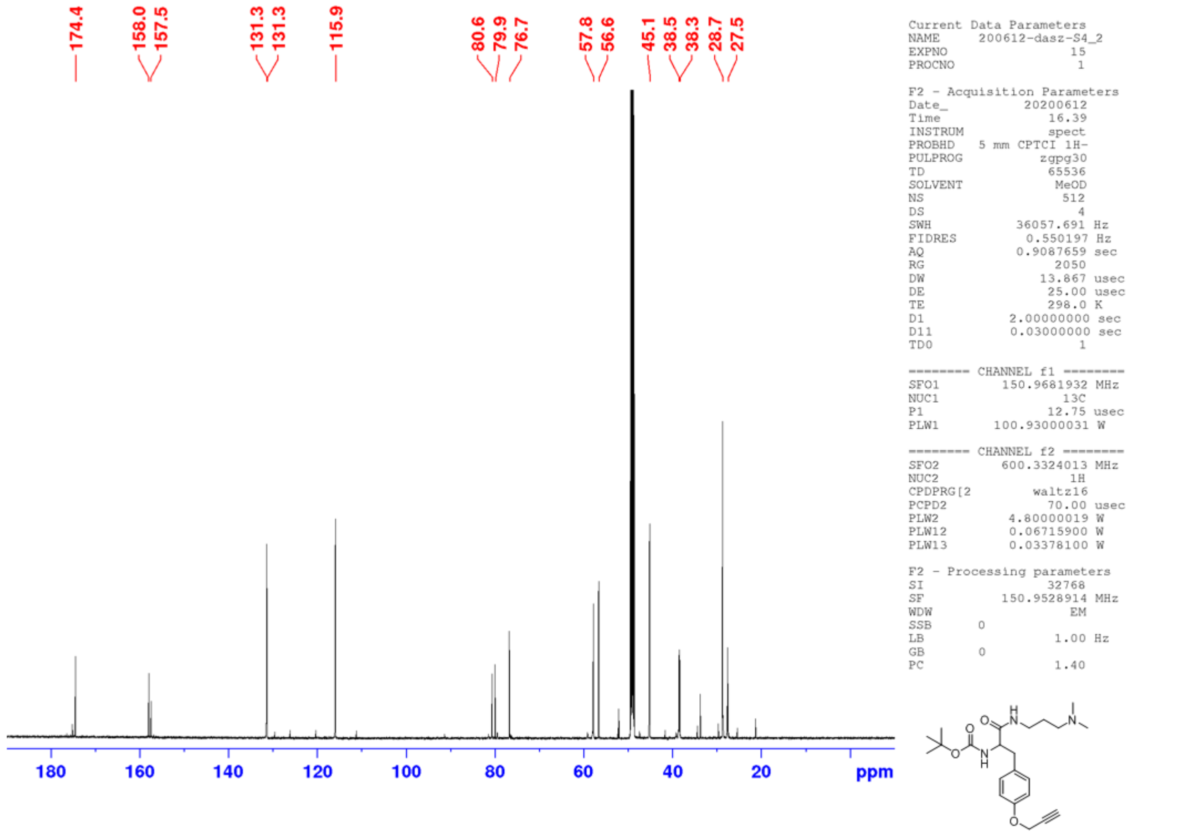

^{13}C-NMR spectrum of **(6)** in MeOD

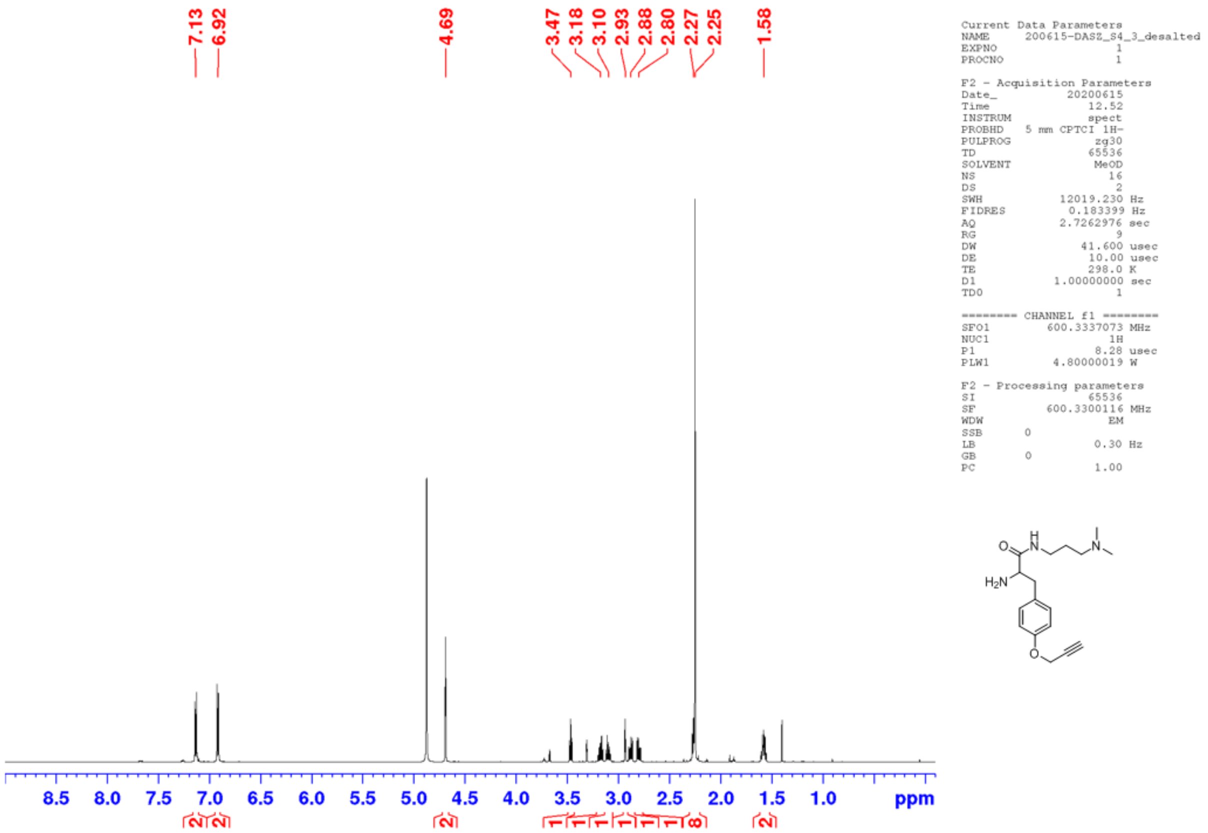

^{1}H-NMR spectrum of **(6b)** in MeOD

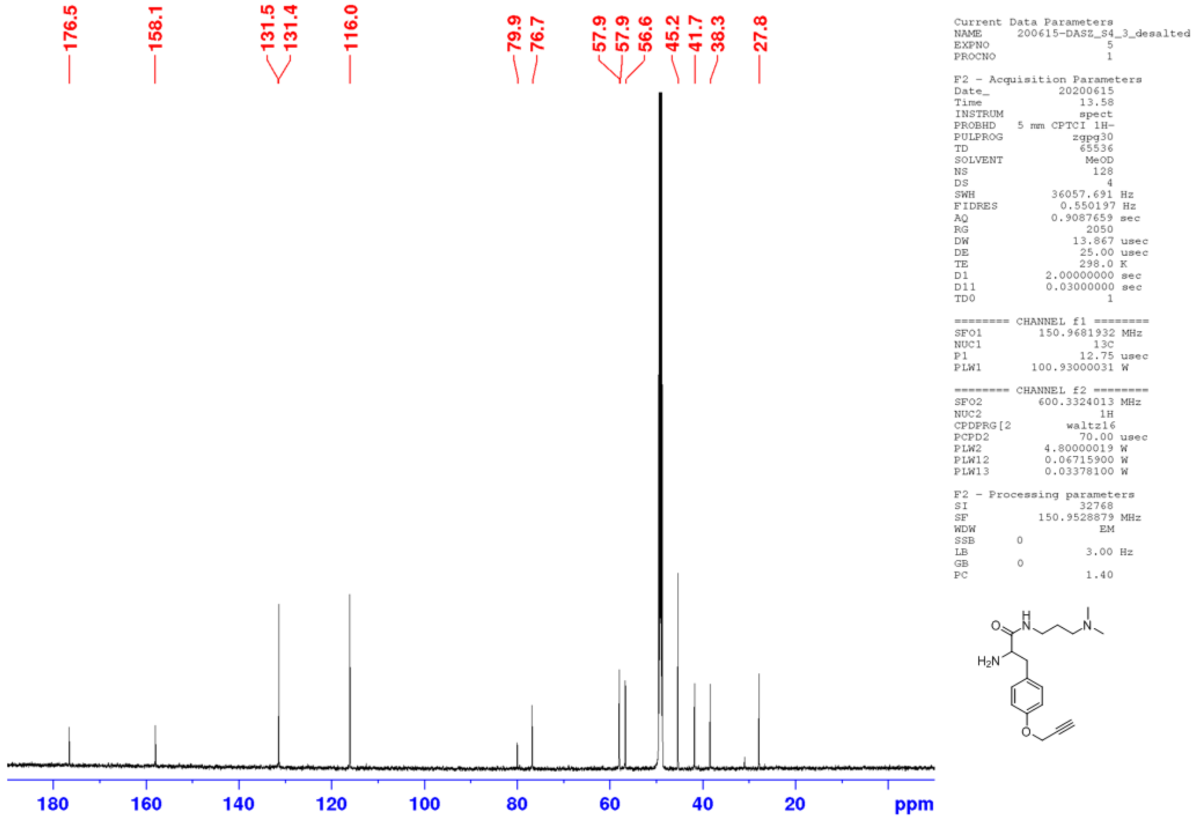

^{13}C-NMR spectrum of **(6b)** in MeOD

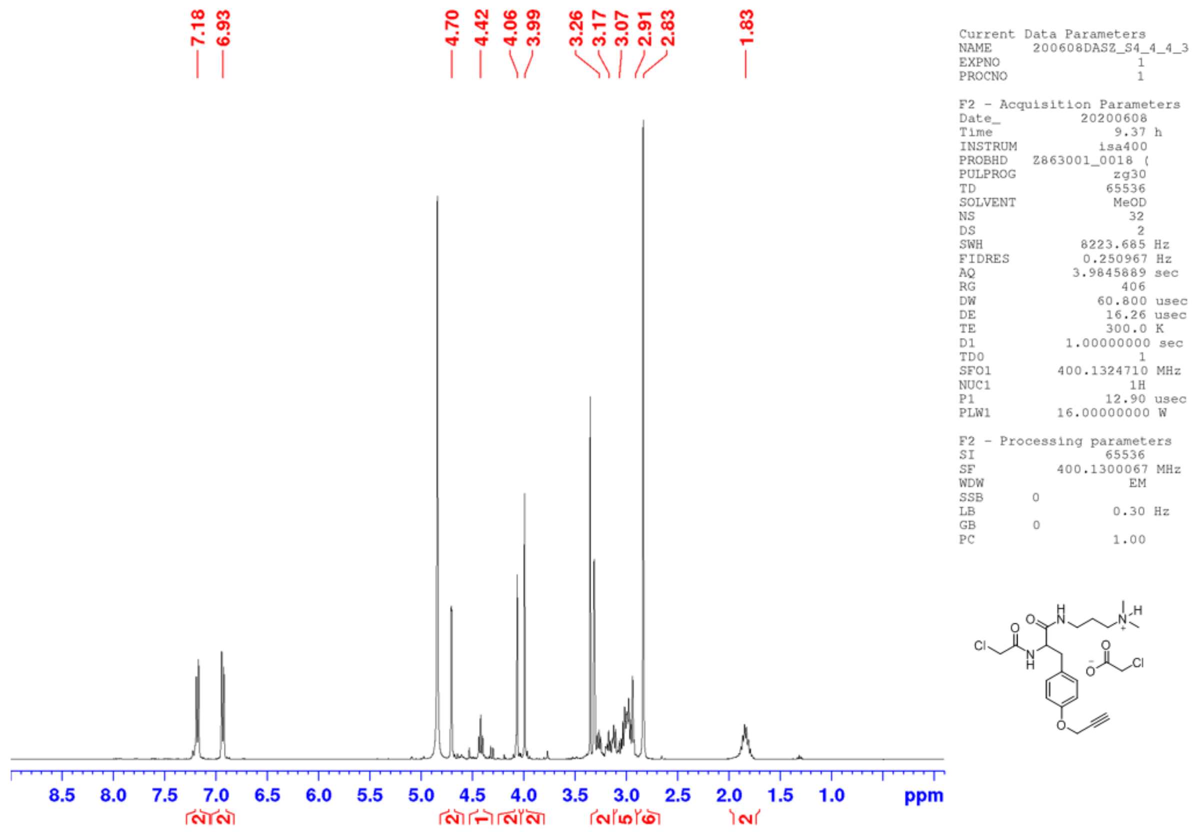

^{1}H-NMR spectra of **(7a)** chloroacetate salt in MeOD

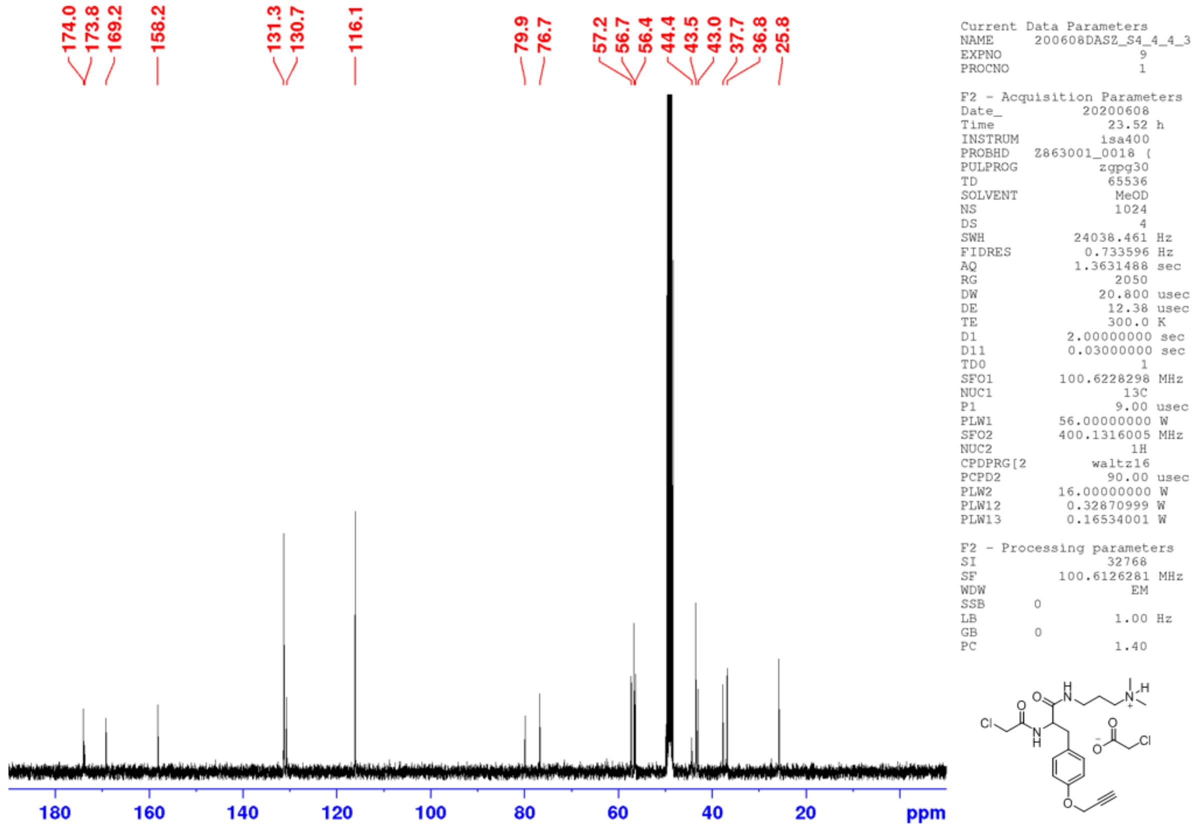

^{13}C-NMR spectra of **(7a)** chloroacetate salt in MeOD

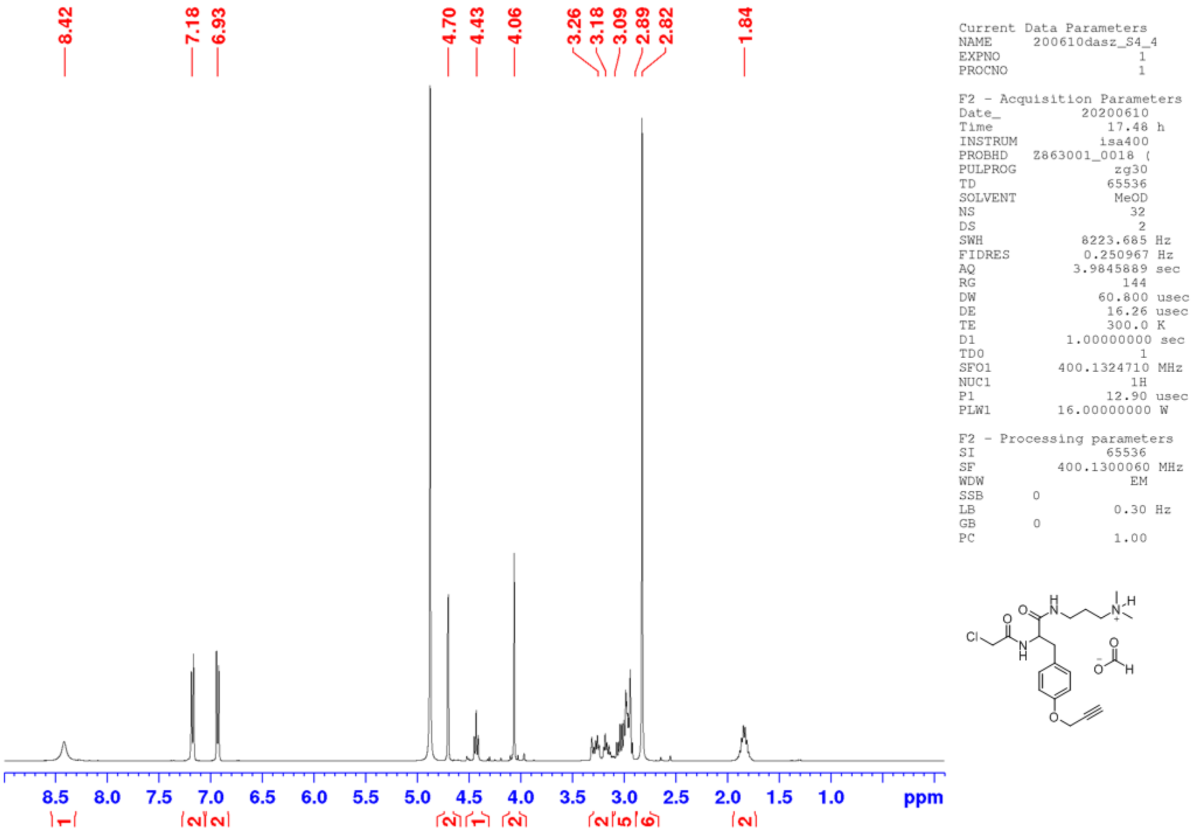

^{1}H-NMR spectra of **(7b)** formate salt in MeOD

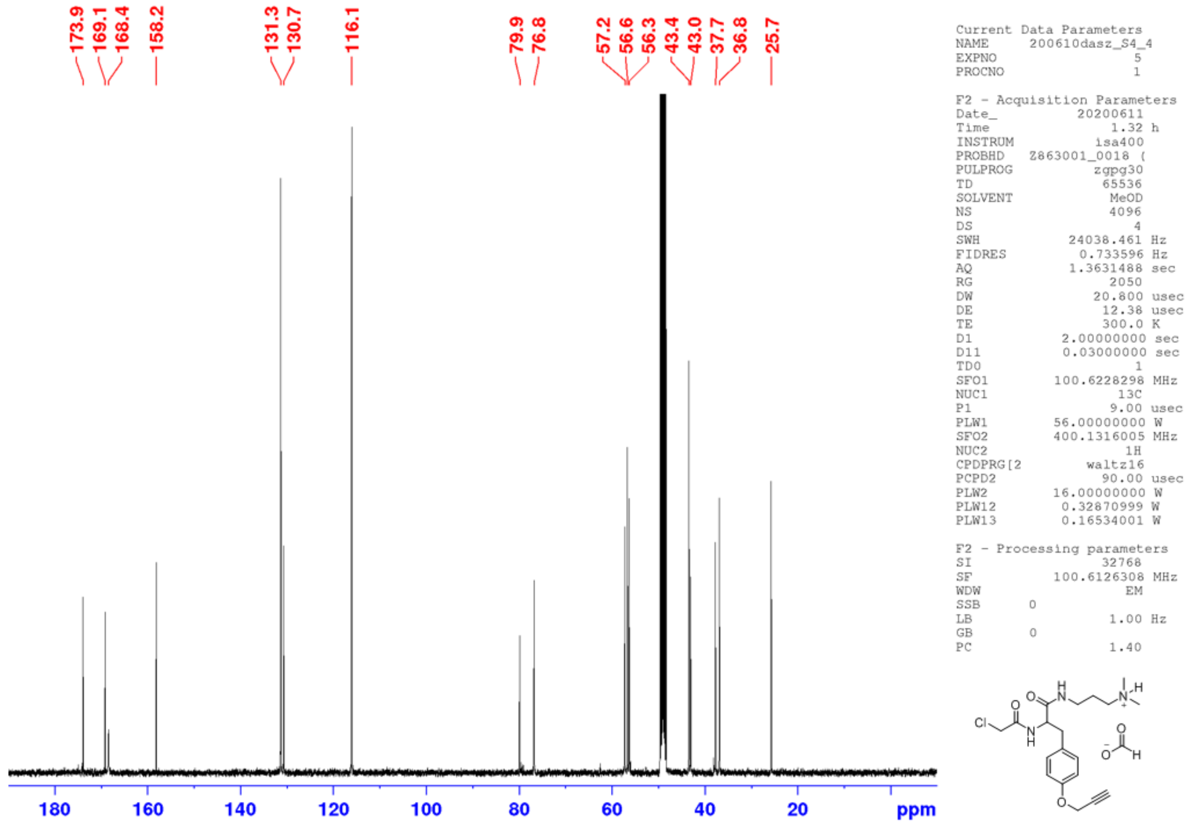

^{13}C-NMR spectra of **(7b)** formate salt in MeOD
